# Association between DNA Methylation in Whole Blood and Measures of Glucose Metabolism: KORA F4 Study

**DOI:** 10.1371/journal.pone.0152314

**Published:** 2016-03-28

**Authors:** Jennifer Kriebel, Christian Herder, Wolfgang Rathmann, Simone Wahl, Sonja Kunze, Sophie Molnos, Nadezda Volkova, Katharina Schramm, Maren Carstensen-Kirberg, Melanie Waldenberger, Christian Gieger, Annette Peters, Thomas Illig, Holger Prokisch, Michael Roden, Harald Grallert

**Affiliations:** 1 Research Unit of Molecular Epidemiology, Helmholtz Zentrum Muenchen, German Research Center for Environmental Health, Neuherberg, Germany; 2 Institute of Epidemiology II, Helmholtz Zentrum Muenchen, German Research Center for Environmental Health, Neuherberg, Germany; 3 German Center for Diabetes Research (DZD), Muenchen-Neuherberg, Germany; 4 Institute for Clinical Diabetology, German Diabetes Center, Leibniz Center for Diabetes Research at Heinrich Heine University Duesseldorf, Duesseldorf, Germany; 5 Institute for Biometrics and Epidemiology, German Diabetes Center, Leibniz Center for Diabetes Research at Heinrich Heine University Duesseldorf, Duesseldorf, Germany; 6 Institute of Human Genetics, Helmholtz Zentrum Muenchen, German Research Center for Environmental Health, Neuherberg, Germany; 7 Institute of Human Genetics, Technische Universitaet Muenchen, Munich, Germany; 8 Hannover Unified Biobank, Hannover Medical School, Hanover, Germany; 9 Institute of Human Genetics, Hannover Medical School, Hanover, Germany; 10 Department of Endocrinology and Diabetology, University Hospital Duesseldorf, Duesseldorf, Germany; CEA - Institut de Genomique, FRANCE

## Abstract

Epigenetic regulation has been postulated to affect glucose metabolism, insulin sensitivity and the risk of type 2 diabetes. Therefore, we performed an epigenome-wide association study for measures of glucose metabolism in whole blood samples of the population-based Cooperative Health Research in the Region of Augsburg F4 study using the Illumina HumanMethylation 450 BeadChip. We identified a total of 31 CpG sites where methylation level was associated with measures of glucose metabolism after adjustment for age, sex, smoking, and estimated white blood cell proportions and correction for multiple testing using the Benjamini-Hochberg (B-H) method (four for fasting glucose, seven for fasting insulin, 25 for homeostasis model assessment-insulin resistance [HOMA-IR]; B-H-adjusted p-values between 9.2x10^-5^ and 0.047). In addition, DNA methylation at cg06500161 (annotated to *ABCG1*) was associated with all the aforementioned phenotypes and 2-hour glucose (B-H-adjusted p-values between 9.2x10^-5^ and 3.0x10^-3^). Methylation status of additional three CpG sites showed an association with fasting insulin only after additional adjustment for body mass index (BMI) (B-H-adjusted p-values = 0.047). Overall, effect strengths were reduced by around 30% after additional adjustment for BMI, suggesting that this variable has an influence on the investigated phenotypes. Furthermore, we found significant associations between methylation status of 21 of the aforementioned CpG sites and 2-hour insulin in a subset of samples with seven significant associations persisting after additional adjustment for BMI. In a subset of 533 participants, methylation of the CpG site cg06500161 (*ABCG1*) was inversely associated with *ABCG1* gene expression (B-H-adjusted p-value = 1.5x10^-9^). Additionally, we observed an enrichment of the top 1,000 CpG sites for diabetes-related canonical pathways using Ingenuity Pathway Analysis. In conclusion, our study indicates that DNA methylation and diabetes-related traits are associated and that these associations are partially BMI-dependent. Furthermore, the interaction of *ABCG1* with glucose metabolism is modulated by epigenetic processes.

## Introduction

Many factors, not only environment and lifestyle, but also genes, contribute to the development of type 2 diabetes (T2D) [1, 2]. The heritability of T2D and related traits has been estimated to be between 15 and 85% [3–7]. So far, 88 genetic susceptibility loci for T2D have been identified. However, these loci explain only 5–10% of the estimated heritability [8–13]. The analysis of DNA methylation patterns is expected to reveal epigenetic modifications associated with T2D at a genome-wide scale and may therefore hopefully help to clarify some of the missing heritability and improve our understanding of the pathomechanisms leading to T2D.

A small number of cross-sectional studies have provided first evidence for an association between DNA methylation and T2D. In skeletal muscle, peroxisome proliferator-activated receptor gamma (PPARgamma) coactivator-1 alpha (PGC-1alpha) is hypermethylated in T2D patients compared to glucose tolerant individuals [14]. Furthermore, increased DNA methylation on the *FTO* obesity susceptibility haplotype was found when comparing human whole blood of 30 diabetic females and 30 females without diabetes [15]. A larger study comprising 710 T2D cases and 459 controls observed an excess of differentially methylated sites in genomic regions that are associated with T2D [16]. However, analyses of genome-wide DNA methylation and T2D-related traits as the phenotypes of interest in a population-based setting using whole blood have not been previously published.

Hallmarks in the pathogenesis of T2D are reduced insulin sensitivity and impaired insulin secretion, which eventually lead to chronic hyperglycemia [17, 18]. Therefore, we aimed to investigate (i) associations between DNA methylation and T2D-related traits [glucose, insulin, HOMA-IR (homeostasis model assessment-insulin resistance)]; (ii) the impact of BMI on these associations; (iii) associations between DNA methylation at the significant CpG sites and gene expression; and (iv) the enrichment for pathways linked to diabetes. To these ends, we performed epigenome-wide association studies (EWAS) using whole blood samples from a population-based prospective study, the Cooperative Health Research in the Region of Augsburg (KORA) F4 study. We provide evidence that the interaction of *ABCG1* with glucose metabolism is modulated by epigenetic processes.

## Material/Methods

### Ethics statement

The study was conducted according to the principles expressed in the Declaration of Helsinki. Written informed consent was obtained from all participants. The study, including the protocol for subject recruitment and assessment and the informed consent for participants, was reviewed and approved by the local ethical committee (Bayerische Landesärztekammer).

### Study population

The KORA studies comprise a series of independent population-based epidemiological surveys and follow-up examinations of individuals living in the region of Augsburg and two adjacent counties in Southern Germany [19]. No evidence for population stratification in the KORA study was found [20].

DNA methylation data were generated for 1,814 participants of the KORA F4 study (2006–2008), who were randomly selected from a total of 3,080 KORA F4 participants aged between 32 and 81 years [21]. Fifteen samples were excluded upon quality control, resulting in 1,799 participants available for our study. In addition, 351 participants were excluded due to exhibiting at least one of the following criteria:

- non-fasting (less than 8 hours),
- overt diabetes (fasting glucose ≥ 7 mmol/l and/or 2-hour glucose ≥ 11.1 mmol/l and/or HbA1c ≥ 6.5% [22] and/or known diabetes and/or diabetes treatment),
- unknown diabetes status as well as unclear information concerning the diabetes status,
- missing values in at least one of the outcome parameters and/or covariates,
- hsC-reactive protein > 10 mg/l (as an indicator for an acute infection),
- participants with fasting insulin ≥ 55.718 [μlU/ml] (corresponding to the 99^th^ percentile) as they clearly represented outliers in the dataset for this variable.

**Fig 1:**
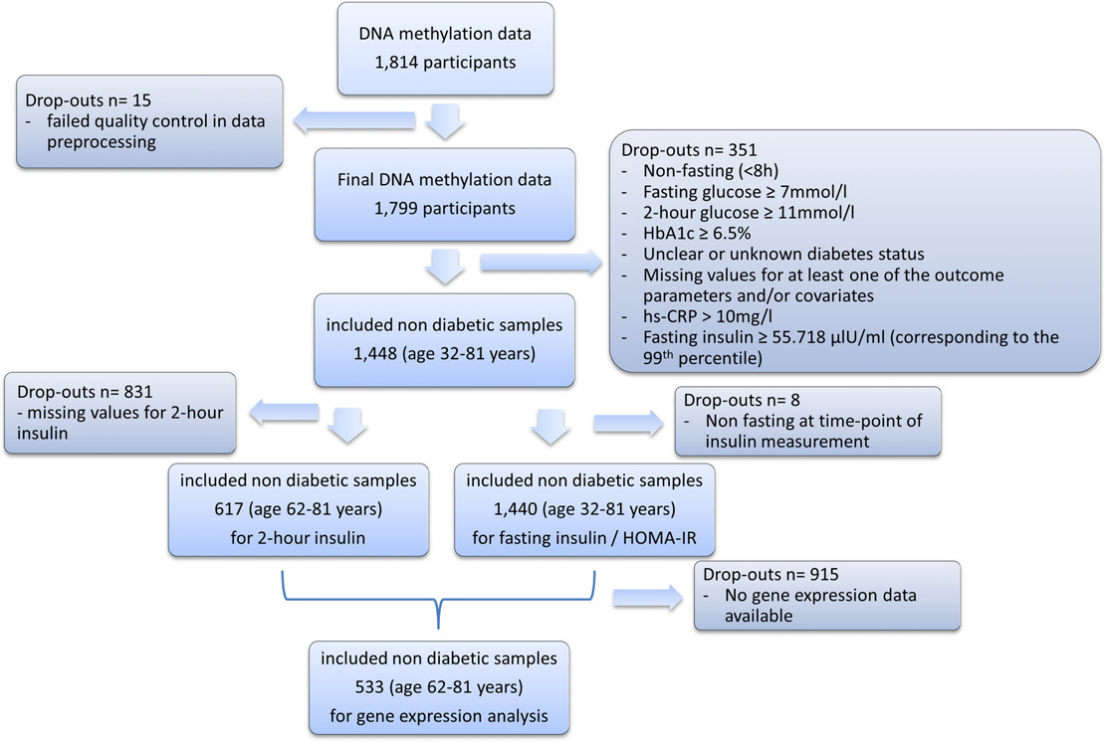
Flow diagram for the study population.

Data from 1,448 non-diabetic individuals aged between 32 and 81 years (see also Table 1) were included in the EWAS of fasting glucose, 2-hour glucose, and HbA1c (Fig 1). For the EWAS of fasting insulin and HOMA-IR and 2-hour insulin, 1,440 (aged 32–81 years) and 617 (aged 62–81 years) participants were included, respectively (S1 and S2 Tables, Fig 1). Gene expression analyses were possible in a subgroup of 533 subjects aged 62 to 81 years for whom both gene expression data and methylation data were available (S3 Table, Fig 1) [23].

**Table 1:** Characteristics of the study population (n = 1,448) for the DNA methylation analyses of fasting glucose, 2-hour glucose, and HbA1c.

	Median (25^th^; 75^th^ percentile)	%
**Sex [% male]**	-	47.1
**Age [years]**	59 (53; 67)	-
**BMI [kg/m**^**2**^**]**	27.1 (24.5; 30.1)	-
**Waist circumference [cm]**	93.5 (84.5; 102.3)	-
**Fasting serum glucose [mmol/l]**	5.3 (4.9; 5.6)	-
**2-hour serum glucose [mmol/l]**	6.0 (5.0; 7.2)	-
**HbA1c [%]**	5.5 (5.2; 5.7)	-
**Glucose tolerance status [%]**		
NGT	-	77.3
IFG	-	5.0
IGT	-	14.4
Combined IFG and IGT	-	3.4
**Fasting insulin [μlU/ml]**	4.1 (2.8; 6.7)	-
**2-hour insulin [μlU/ml]**	50.2 (28.7; 412.0)	-
**HOMA-IR**	1.0 (0.6; 1.6)	-
**C-reactive protein [mg/l]**	1.2 (0.6; 2.2)	-
**Leucocytes [/nl]**	5.5 (4.7; 6.5)	-
**Total cholesterol [mmol/l]**	5.7 (5.1; 6.4)	-
**Triglycerides [mmol/l]**	1.2 (0.9; 1.7)	-
**Systolic blood pressure [mmHg]**	122.2 (111.0; 134.5)	-
**Diastolic blood pressure [mmHg]**	75.5 (69.5; 82.5)	-
**Alcohol consumption [g/day]**	8.4 (0.0; 22.9)	-
**Smoking status [%]**		
never	-	44.8
ex	-	40.1
current	-	15.1
**Physically active [%]**	-	
(combination of activity during summer and winter with ≥ 1 hour per week)	-	60.2

NGT: normal glucose tolerance. IFG: impaired fasting glucose. IGT: impaired glucose tolerance.

### Assessment of diabetes-related traits

For the determination of fasting glucose and 2-hour glucose levels, fasting venous blood and blood samples after an oral glucose tolerance test (OGTT) for all non-diabetic participants were collected and analyzed as described elsewhere [24–26]. HbA1c values in blood were determined using high performance liquid chromatography (HPLC) (Menarini HA-8160) [24]. Fasting insulin was measured by ELISA [27]. HOMA-IR was calculated as [fasting plasma glucose (mmol/l) x fasting serum insulin (mU/l) / 22.5] [25]. Diabetes status was determined in all surveys via self-report. Self-reports were subsequently validated by contacting the treating physician or additionally performing an OGTT [24].

### Genome-wide DNA methylation analysis

Genomic DNA (1 µg), isolated from whole blood, was bisulfite converted using the EZ-96 DNA Methylation Kit (Zymo Research, Orange, CA, USA) as described recently [21]. Genome-wide DNA methylation was investigated using the Illumina HumanMethylation 450 BeadChip (Illumina, San Diego, CA, USA) following the Illumina Infinium HD Methylation instructions as described [21]. This array comprises >485,000 CpG sites covering 99% of the genes in the reference sequence database [28]. Probes are distributed over the whole gene, including the promotor region, gene body, 3`UTR, and intergenic region [28, 29]. Beta values representing continuous numbers between 0 and 1 reflecting the methylation degree were exported and used for statistical analysis, since methylation in this study is considered as the independent variable. GenomeStudio (version 2010.3) with Methylation Module (version 1.8.5) was used to process the raw image data generated by the BeadArray Reader. Initial quality control of assay performance was undertaken using “Control Dashboard” provided by GenomeStudio Software, including the assessment of staining, extension, hybridization, target removal, bisulfite conversion, specificity, negative, and non-polymorphic control.

### Gene expression studies

Total RNA was extracted from whole blood taken under fasting conditions according to the manufacturer’s instructions using the PAXgene Blood miRNA Kit (QIAGEN, Redwood City, CA, USA). Gene expression profiling was done using the Illumina Human HT-12 v3 Expression BeadChip (Illumina, San Diego, CA, USA) as described elsewhere [23, 30].

### Statistical analysis

The methylation data were preprocessed and quality controlled [21]. The CpG site-wise and sample-wise detection rates were set to 95%. The data were beta-mixture quantile normalized [31] with default parameter settings to avoid bias in the analysis due to the fact that two different chemistries are included on the chip [28]. Prior to modelling, the values of fasting glucose, 2-hour glucose, fasting insulin, and HOMA-IR were log transformed to achieve approximately normal distributions. Epigenome-wide associations between DNA methylation and measures of glucose metabolism (fasting glucose, 2-hour glucose, fasting insulin, HOMA-IR, and HbA1c) were assessed using linear mixed effects models (R package nlme), accounting for plate effects; see Table 2 for lists of covariates. Estimated white blood cell proportions were determined using the method published by Houseman *et al*. [32].

**Table 2:** Regression models for the analysis of association between DNA methylation and measures of glucose metabolism.

Model	Covariates
**M1**	age, sex, estimated white blood cell proportions, smoking
**M2**	age, sex, estimated white blood cell proportions, smoking, BMI

Furthermore, 2-hour insulin, cubic root transformed, was analyzed similarly for those CpG sites whose methylation levels showed genome-wide significance in model 1 for at least one of the phenotypes mentioned above, due to the smaller number of samples with available 2-hour insulin data. All results were corrected for multiple testing using the Benjamini-Hochberg (B-H) method [33]. We checked whether the detected CpG site contained SNPs with MAF ≥0.05 within the probe-binding regions. Cg09349128, cg15309457 and cg27434584 has to be regarded with caution as SNPs were included in the probe-binding region, which could lead to an influence of probe binding. Cross-reactive probes were checked using the list provided by Chen *et al*. [34]. For sensitivity analyses the study population was stratified according to DNA methylation quintiles for the CpG sites whose methylation levels showed significant associations with our phenotypes of interest, and p-values were determined using linear regression for continuous variables and Χ^2^ tests for categorical variables.

For a subset of 533 participants both methylation and gene expression data were available. In this subset the associations between methylation at the significant CpGs and gene expression at nearby genes were analyzed using linear regression models. Transcript probes that mapped to a ±500kb window around the CpG site were included. Models were adjusted for the gene expression specific technical variables [23] sample storage time, RNA integrity number (RIN), and RNA amplification batch, as well as for the methylation specific variables age, sex, BMI, smoking, estimated white blood cell proportions [32], and plate. We accounted for multiple testing using the B-H correction. For methylation as well as gene expression analysis results were defined as significant if the B-H-adjusted p-value was <0.05. All statistical analyses were performed using R (version 2.15.3 or higher).

### Pathway analysis

The Ingenuity Pathway Analysis (IPA) software (IPA build version: 364062M, content version: 26127183, release date: 2015-12-12, analysis date: 2016-01-08, http://www.ingenuity.com/) (QIAGEN, Redwood City, CA, USA) was used to detect potential pathways and networks in the DNA methylation data relevant in the cross-sectional study in an unbiased way. We included the 1,000 CpG sites with the smallest p-values for association with the respective traits and conducted pathway analysis separately for each trait. The database underlying IPA is referred to as the Ingenuity Knowledge Base (Gene and Endogenous Chemicals). The reference set was restricted to genes represented on the Illumina HT-12 v4 BeadChip, and only human annotations were considered. Pathway analyses were performed with IPA’s Core Analysis module. Canonical pathways with a p-value <0.05 after B-H correction were defined as a statistically significant overrepresentation of input genes in a given process.

## Results

### Study population

Characteristics of the main study population of 1,448 individuals (for the analyses of fasting glucose, 2-hour glucose and HbA1c) are provided in Table 1. S1 and S2 Tables give the corresponding information for the subgroups of 1,440 and 617 individuals for the analyses of fasting insulin/HOMA-IR and 2-hour insulin, respectively.

### Association between DNA methylation and diabetes-related traits

**Fig 2:**
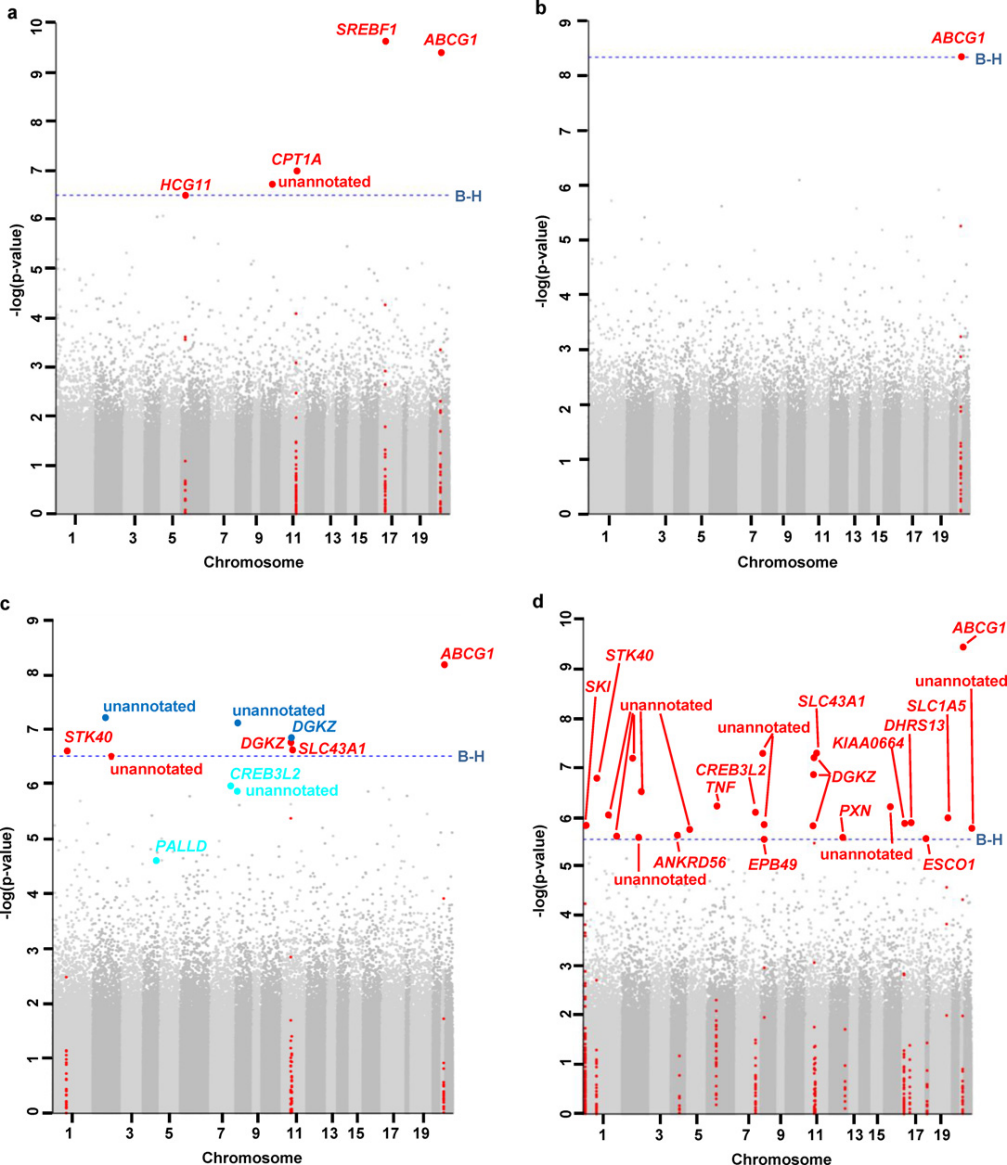
Genome-wide associations between methylation and (a) fasting glucose, (b) 2-hour glucose, (c) fasting insulin, (d) HOMA-IR, after adjustment for sex, age, and estimated white blood cell proportions (model 1). Results are plotted for each chromosome (excluding the sex chromosomes) (x-axis) against the–log (unadjusted p-value) (y-axis). The Benjamini-Hochberg method was used as correction for multiple testing. The dotted line marks the significance threshold. Red dots: methylation levels of these CpG sites are associated with these phenotypes in model 1; turquois dots: methylation levels of these CpG sites are significantly associated with the phenotype after additional adjustment for BMI (model 2); blue dots: methylation levels of these CpG sites are significantly associated with the phenotype in both adjustment models.

In total, methylation levels of 31 CpG sites showed genome-wide significant associations with measures of glucose metabolism (Table 3). Cg09349128, cg15309457 and cg27434584 have to be regarded with caution as SNPs were included in the probe-binding region with MAF > 0.05, which could lead to an influence of probe binding. Methylation at five CpG sites showed genome-wide significant associations with fasting glucose in model 1 (B-H-adjusted p-values between 9.2x10^-5^ and 0.029) (Fig 2A). Results for cg22040809 (*HCG11*) have to be interpreted with caution as this site is listed by Chen *et al*. [34] as a cross-reactive probe, i.e. one that binds to another genomic sequence due to large sequence homology, the resulting signal therefore representing a mixture of methylation levels at different sites. One CpG site was associated with 2-hour glucose in model 1 (B-H-adjusted p-value 2.0x10^-3^) (Fig 2B). The methylation levels of eight CpG sites were associated with fasting insulin in model 1 (B-H-adjusted p-values between 3.0x10^-3^ and 0.017) (Fig 2C). HOMA-IR was associated with methylation status of 26 CpG sites in model 1 (B-H-adjusted p-values between 1.7x10^-4^ and 0.048) (Fig 2D). No genome-wide significant associations could be observed for HbA1c (data not shown). When we tested the association of all aforementioned CpG sites with 2-hour insulin, we observed significant associations for 21 CpG sites in model 1 (B-H-adjusted p-values between 9.0x10^-6^ and 0.042). In addition, we observed suggestive evidence for associations of CpG sites with fasting insulin (B-H-adjusted p-values 0.051) (Table 3).

**Table 3:** Results for associations between genome-wide DNA methylation levels and fasting glucose, 2-hour glucose, fasting insulin, 2-hour insulin, and HOMA-IR, after adjustment for different potential confounders.

						Age, sex, estimated white blood cell proportions, smoking (model 1)			Age, sex, estimated white blood cell proportions, BMI, smoking (model 2)		
Phenotype	CpG	Annotated gene	Location on chromosome	Location within gene	Relation to CpG island	Coeffi cient	p-value	B-H-adjusted p-value	Coeffi cient	p-value	B-H-adjusted p-value
**Fasting glucose**	cg00574958	*CPT1A*	11:68607622	5'UTR	N Shore	**-0.097**	**9.8x10**^**-8**^	**0.014**	-0.060	5.8x10^-4^	0.985
	cg06500161	*ABCG1*	21:43656587	gene body	S Shore	**0.044**	**4.2x10**^**-10**^	**9.2x10**^**-5**^	0.026	1.2x10^-4^	0.833
	cg07504977	unannotated	10:102131012	-	N Shelf	**0.023**	**2.0x10**^**-7**^	**0.022**	0.017	5.5x10^-5^	0.712
	cg11024682	*SREBF1*	17:17730094	gene body	S Shelf	**0.056**	**2.5x10**^**-10**^	**9.2x10**^**-5**^	0.041	2.0x10^-6^	0.569
	cg22040809^#^	*HCG11*	6:26522578	gene body	Island	**-0.021**	**3.2x10**^**-7**^	**0.029**	-0.018	9.6x10^-6^	0.627
**2-hour glucose**	cg06500161	*ABCG1*	21:43656587	gene body	S Shore	**0.042**	**4.6x10**^**-9**^	**2.0x10**^**-3**^	0.027	1.6x10^-4^	0.981
Fasting insulin*	cg03581271	*PALLD*	4:169806766	gene body	-	0.032	2.5x10^-5^	0.183	**0.035**	**5.2x10**^**-7**^	**0.047**
	cg06500161	*ABCG1*	21:43656587	gene body	S Shore	**0.043**	**6.9x10**^**-9**^	**3.0x10**^**-3**^	0.018	8.6x10^-3^	0.373
	cg07092212	*DGKZ*	11:46382544	gene body, TSS1500^$^	-	**-0.080**	**1.7x10**^**-7**^	**0.015**	-0.066	2.8x10^-6^	0.111
	cg09613192	unannotated	2:181388538	-	-	**0.021**	**3.0x10**^**-7**^	**0.017**	0.013	4.7x10^-4^	0.331
	cg09694782	unannotated	2:97408799	-	S Shelf	**-0.037**	**5.8x10**^**-8**^	**0.011**	**-0.031**	**4.7x10**^**-7**^	**0.047**
	cg11376147	*SLC43A1*	11:57261198	gene body	-	**-0.076**	**2.1x10**^**-7**^	**0.015**	-0.041	2.3x10^-3^	0.340
	cg13016916	*CREB3L2*	7:137660322	gene body	-	0.016	1.2x10^-6^	0.051	**0.015**	**5.1x10**^**-7**^	**0.047**
	cg17266233	*DGKZ*	11:46382725	gene body, TSS1500^$^	-	**-0.102**	**1.6x10**^**-7**^	**0.015**	**-0.088**	**6.3x10**^**-7**^	**0.047**
	cg17971578	*STK40*	1:36852463	TSS1500	S Shore	**-0.039**	**2.7x10**^**-7**^	**0.017**	-0.019	5.5x10^-3^	0.356
	cg22065733	unannotated	8:11801320	-	-	**-0.067**	**7.2x10**^**-8**^	**0.011**	**-0.057**	**5.3x10**^**-7**^	**0.047**
	cg23899654	unannotated	8:11759930	-	Island	-0.073	1.4x10^-6^	0.051	**-0.068**	**6.1x10**^**-7**^	**0.047**
2-hour insulin***	cg00574958	*CPT1A*	11:68607622	5'UTR	N_Shore	**-0.101**	**8.1x10**^**-4**^	**3.4x10**^**-3**^	-0.063	0.030	0.089
	cg01751800	*ANKRD56*	4:77820391	TSS1500	S_Shore	**-0.017**	**0.016**	**0.031**	-0.014	0.032	0.089
	cg02711608	*SLC1A5*	19:47287964	1st Exon, 5'UTR, gene body	N_Shelf	**-0.025**	**0.018**	**0.031**	-0.010	0.296	0.417
	cg03979241	*EPB49*	8:21916824	1st Exon, 5'UTR^$^	S_Shelf	**0.044**	**9.0x10**^**-4**^	**3.4x10**^**-3**^	**0.042**	**6.0x10**^**-4**^	**9.4x10**^**-3**^
	cg04161365	*DHRS13*	17:27230393	TSS1500	S_Shore	**-0.030**	**0.016**	**0.031**	-0.018	0.120	0.196
	cg06500161	*ABCG1*	21:43656587	gene body	S_Shore	**0.058**	**2.9x10**^**-7**^	**9.0x10**^**-6**^	**0.040**	**2.0x10**^**-4**^	**6.1x10**^**-3**^
	cg06946797	unannotated	16:11422409	-	-	**-0.032**	**1.5x10**^**-4**^	**2.3x10**^**-3**^	**-0.025**	**1.4x10**^**-3**^	**0.012**
	cg07504977	unannotated	10:102131012	-	N_Shelf	**0.015**	**0.029**	**0.042**	0.010	0.106	0.196
	cg09469355	*SKI*	1:2161886	gene body	S_Shore	**-0.036**	**9.8x10**^**-4**^	**3.4x10**^**-3**^	-0.020	0.059	0.141
	cg09694782	unannotated	2:97408799	-	S_Shelf	**-0.036**	**6.9x10**^**-4**^	**3.4x10**^**-3**^	**-0.031**	**1.6x10**^**-3**^	**0.012**
	cg11024682	*SREBF1*	17:17730094	gene body	S_Shelf	**0.043**	**2.9x10**^**-3**^	**8.8x10**^**-3**^	0.031	0.022	0.076
	cg11307565	*PXN*	12:120701165	gene body	N_Shore	**-0.046**	**4.6x10**^**-4**^	**3.4x10**^**-3**^	**-0.036**	**3.4x10**^**-3**^	**0.021**
	cg11376147	*SLC43A1*	11:57261198	gene body	-	**-0.077**	**7.4x10**^**-4**^	**3.4x10**^**-3**^	-0.046	0.036	0.094
	cg11990813	KIAA0664	17:2603739	gene body	S_Shore	**0.067**	**3.5x10**^**-3**^	**9.1x10**^**-3**^	**0.058**	**7.7x10**^**-3**^	**0.034**
	cg12593793	unannotated	1:156074135	-	-	**-0.037**	**6.7x10**^**-3**^	**0.016**	-0.010	0.439	0.545
	cg13016916	*CREB3L2*	7:137660322	gene body	-	**0.015**	**3.1x10**^**-3**^	**8.8x10**^**-3**^	**0.013**	**4.0x10**^**-3**^	**0.021**
	cg15309457*	unannotated	5:1746469	-	Island	**-0.018**	**0.027**	**0.042**	-0.005	0.498	0.559
	cg17588294	unannotated	2:129548156	-	-	**-0.031**	**0.012**	**0.026**	-0.020	0.086	0.177
	cg17971578	*STK40*	1:36852463	TSS1500	S_Shore	**-0.044**	**5.0x10**^**-4**^	**3.4x10**^**-3**^	-0.022	0.067	0.147
	cg20477259	*TNF*	6:31544960	gene body	N_Shelf	**-0.041**	**0.017**	**0.031**	-0.015	0.378	0.509
	cg27434584*	*ESCO1*	18:19114052	gene body	-	**0.032**	**0.022**	**0.037**	0.032	0.015	0.057
HOMA-IR**	cg01751800	*ANKRD56*	4:77820391	TSS1500	S Shore	**-0.022**	**2.5x10**^**-6**^	**0.047**	-0.017	8.5x10^-5^	0.259
	cg02711608	*SLC1A5*	19:47287964	1st Exon, 5'UTR, gene body^$^	N Shelf	**-0.033**	**1.1x10**^**-6**^	**0.037**	-0.021	5.0x10^-4^	0.294
	cg03979241	*EPB49*	8:21916824	1st Exon, 5'UTR^$^	S Shelf	**0.037**	**2.8x10**^**-6**^	**0.048**	0.030	3.2x10^-5^	0.208
	cg04161365	*DHRS13*	17:27230393	TSS1500	S Shore	**-0.040**	**1.3x10**^**-6**^	**0.038**	-0.028	1.4x10^-4^	0.273
	cg06500161	*ABCG1*	21:43656587	gene body	S Shore	**0.047**	**3.9x10**^**-10**^	**1.7x10**^**-4**^	0.021	2.7x10^-3^	0.314
	cg06946797	unannotated	16:11422409	-	-	**-0.027**	**6.6x10**^**-7**^	**0.029**	-0.017	3.3x10^-4^	0.287
	cg07092212	*DGKZ*	11:46382544	gene body, TSS1500^$^	-	**-0.083**	**5.6x10**^**-8**^	**5.6x10**^**-3**^	-0.067	9.5x10^-7^	0.057
	cg09349128*	unannotated	22:50327986	-	N Shore	**-0.036**	**1.8x10**^**-6**^	**0.042**	-0.019	7.7x10^-3^	0.342
	cg09469355	*SKI*	1:2161886	gene body	S Shore	**-0.034**	**1.6x10**^**-6**^	**0.040**	-0.019	3.1x10^-3^	0.316
	cg09613192	unannotated	2:181388538	-	-	**0.021**	**3.4x10**^**-7**^	**0.019**	0.013	6.1x10^-4^	0.294
	cg09694782	unannotated	2:97408799	-	S Shelf	**-0.037**	**6.4x10**^**-8**^	**5.6x10**^**-3**^	-0.031	5.1x10^-7^	0.051
	cg11307565	*PXN*	12:120701165	gene body	N Shore	**-0.040**	**2.8x10**^**-6**^	**0.048**	-0.029	2.4x10^-4^	0.287
	cg11376147	*SLC43A1*	11:57261198	gene body	-	**-0.079**	**5.1x10**^**-8**^	**5.6x10**^**-3**^	-0.043	1.2x10^-3^	0.306
	cg11990813	*KIAA0664*	17:2603739	gene body	S Shore	**0.071**	**1.4x10**^**-6**^	**0.038**	0.059	6.7x10^-6^	0.149
	cg12593793	unannotated	1:156074135	-	-	**-0.042**	**1.0x10**^**-6**^	**0.037**	-0.011	0.166	0.595
	cg13016916	*CREB3L2*	7:137660322	gene body	-	**0.016**	**8.7x10**^**-7**^	**0.035**	0.015	3.1x10^-7^	0.051
	cg15309457*	unannotated	5:1746469	-	Island	**-0.025**	**1.9x10**^**-6**^	**0.042**	-0.017	5.5x10^-4^	0.294
	cg17266233	*DGKZ*	11:46382725	gene body, TSS1500^$^	-	**-0.102**	**1.4x10**^**-7**^	**0.010**	-0.087	5.7x10^-7^	0.051
	cg17588294	unannotated	2:129548156	-	-	**-0.038**	**2.5x10**^**-6**^	**0.047**	-0.027	1.8x10^-4^	0.287
	cg17971578	*STK40*	1:36852463	TSS1500	S Shore	**-0.039**	**1.8x10**^**-7**^	**0.011**	-0.019	5.7x10^-3^	0.334
	cg18337963	*DGKZ*	11:46383209	1st Exon, 5'UTR, gene body^$^	-	**-0.069**	**1.5x10**^**-6**^	**0.038**	-0.063	1.3x10^-6^	0.057
	cg20477259	*TNF*	6:31544960	gene body	N Shelf	**-0.055**	**6.5x10**^**-7**^	**0.029**	-0.031	2.6x10^-3^	0.313
	cg22065733	unannotated	8:11801320	-	-	**-0.067**	**5.2x10**^**-8**^	**5.6x10**^**-3**^	-0.057	3.8x10^-7^	0.051
	cg22798214	unannotated	1:212692190	-	S Shelf	**-0.035**	**2.4x10**^**-6**^	**0.047**	-0.024	4.3x10^-4^	0.294
	cg23899654	unannotated	8:11759930	-	Island	**-0.072**	**1.4x10**^**-6**^	**0.038**	-0.068	5.6x10^-7^	0.051
	cg27434584*	*ESCO1*	18:19114052	gene body	-	**0.041**	**2.6x10**^**-6**^	**0.047**	0.038	1.3x10^-6^	0.057

Results are presented with regression coefficients (increase in SD of phenotype per 1% increase in methylation), unadjusted p-values und B-H-adjusted p-values. Chromosome information is based on Genome Build 37.

^#^ CpG site has to be regarded with caution as it is listed as a cross-reactive probe by Chen *et al*. [34].

* CpG site has to be regarded with caution as SNPs were included in the probe-binding region, which could lead to an influence of probe binding.

** Analyses for fasting insulin and HOMA-IR were performed with a reduced data set (n = 1,440).

*** Analyses for 2-hour insulin were performed with a reduced data set (n = 617) and only for CpG sites (n = 31) whose methylation levels were significantly associated with the other investigated phenotypes. TSS: Transcription start site.

^$^ depending on transcription variant. CpG island: site with an accumulation of CpG sites. shore: located next to the CpG island. shelf: located more than 2kb away from the CpG island. S/N forward-space of shore or shelf: specifies the location in relation to the CpG island (N upstream the CpG island; S downstream the CpG site).

### Impact of additional adjustment for BMI on associations between DNA methylation and diabetes-related traits

The methylation status of three CpG sites was still significantly associated with fasting insulin after additional adjustment for BMI in model 2 (B-H-adjusted p-values 0.047). Methylation of three additional CpG sites was found to be significant with this phenotype only in model 2 (Table 3, Fig 2C). Methylation at seven CpG sites remained significantly associated with 2-hour insulin after adjustment for BMI in model 2 (Table 3). In addition, we observed suggestive evidence for associations of some CpG sites with 2-hour insulin and HOMA-IR (B-H-adjusted p-values between 0.051 and 0.076) (Table 3).

Comparing the coefficients in model 1 vs model 2 for each CpG site separately, the effect strengths were reduced on average by 29.2% (range 14.3–40.9%) for fasting glucose, suggesting a confounding or mediating effect of BMI. Furthermore, effect strengths were reduced by 36.1% for 2-hour glucose through adjustment for BMI, for fasting insulin on average by 23.6% (range 6.3–58.1%), for 2-hour insulin on average by 33.4% (range 3.2–72.5%), and for HOMA-IR on average by 29.8% (range 5.6–73.8%).

### Distribution of metabolic variables in different DNA methylation quintiles

Next, we explored the relation between clinical phenotypes and methylation levels stratified into quintiles at eleven CpG sites significantly associated with fasting insulin and 2-hour insulin in model 2 [cg03581271 (*PALLD*), cg17266233 (*DGKZ*), cg22065733 (unannotated), cg23899654 (unannotated), cg03979241 (*EPB49*), cg06500161 (*ABCG1*), cg06946797 (unannotated), cg09694782 (unannotated), cg11307565 (*PXN*), cg11990813 (*KIAA0064*), and cg13016916 (*CREB3L2*)]. For cg06500161 (*ABCG1*) we observed significant associations between methylation and anthropometric as well as metabolic variables (Table 4): the strongest associations were with waist circumference, triglycerides, fasting glucose, and BMI (p-values for trend <10^−20^). In addition, strong associations between methylation and 2-hour glucose, CD8^+^ T cells, and monocytes (the last two being estimated quantities) (p-values for trend 10^−11^–10^−13^) were observed. For cg09694782 significant associations were observed with age, fasting insulin, and HOMA-IR, as well as for all estimated white blood cell proportions (S4 Table). For cg13016916 no significant associations could only be detected (S5 Table). For cg11990813 significant associations were found for fasting insulin, HOMA-IR, CD8^+^ T cells, CD4^+^ T cells, natural killer cells, and granulocytes (S6 Table). For cg11307565 significant associations were detected for BMI, fasting glucose, fasting insulin, HOMA-IR, CD8^+^ T cells, CD4^+^ T cells, natural killer cells, and granulocytes (S7 Table). For cg06946797 significant associations were observed for age, BMI, waist circumference, C-reactive protein, fasting insulin, 2-hour insulin, HOMA-IR, and almost all estimated white blood cell proportions (S8 Table). For cg03979241 a strong association could be detected for CD8^+^ T cells, CD4^+^ T cells, B cells, and granulocytes besides age, sex, and systolic blood pressure (S9 Table). For cg23899654 significant associations with 2-hour glucose, CD8^+^ T cells and granulocytes were observed (S10 Table). For cg22065733 significant associations with CD8^+^ T cells and granulocytes were found (S11 Table). For cg17266233 significant associations for the estimated white blood cell proportion of CD8^+^ T cells, B cells and granulocytes were detected (S12 Table) and for cg03581271 with fasting insulin (S13 Table).

**Table 4:** Associations between DNA methylation at cg06500161 (*ABCG1*) and different phenotypes, based on quintiles of methylation level.

	Quintile 1 (n = 290)	Quintile 2 (n = 289)	Quintile 3 (n = 290)	Quintile 4 (n = 289)	Quintile 5 (n = 290)	
Continuous phenotype	Mean (SD)	Mean (SD)	Mean (SD)	Mean (SD)	Mean (SD)	p for trend (Bonf. adjusted)
Age [years] #	58.22 (8.77)	58.95 (8.44)	60.50 (8.46)	60.13 (8.53)	61.47 (9.10)	2.21x10^-5^
BMI [kg/m^2^] #	26.01 (3.96)	27.09 (4.28)	27.13 (3.99)	28.10 (4.41)	29.29 (4.45)	8.30x10^-21^
Waist circumference [cm]	87.39 (12.33)	92.04 (12.21)	92.29 (11.27)	95.93 (12.53)	100.60 (12.42)	1.20x10^-38^
Fasting glucose [mmol/l] #	5.12 (0.47)	5.24 (0.48)	5.32 (0.53)	5.36 (0.52)	5.52 (0.55)	4.80x10^-21^
2-hour glucose [mmol/l] #	5.71 (1.53)	6.16 (1.70)	6.15 (1.72)	6.28 (1.67)	6.80 (1.75)	5.82x10^-13^
HbA1c [%]	5.41 (0.33)	5.45 (0.32)	5.45 (0.29)	5.46 (0.32)	5.57 (0.31)	1.70x10^-7^
C-reactive protein [mg/l] #	1.68 (1.76)	1.61 (1.57)	1.70 (1.57)	1.80 (1.74)	1.84 (1.67)	1
Fasting insulin [μlU/ml] # ^1^	5.03 (6.03)	6.17 (7.33)	5.71(5.50)	6.47 (6.24)	7.92 (7.85)	4.94x10^-6^
2-hour insulin [μlU/ml] # ^2^	52.55 (43.42)	54.19 (41.80)	57.59 (41.08)	66.07 (50.52)	81.47 (66.03)	7.66x10^-6^
HOMA-IR # ^1^	1.17 (1.56)	1.47 (1.78)	1.39 (1.44)	1.58 (1.63)	2.00 (2.16)	1.93x10^-7^
Cholesterol [mmol/l] #	5.90 (0.97)	5.85 (0.97)	5.79 (0.99)	5.83 (1.02)	5.63 (1.06)	0.030
Triglycerides [mmol/l] #	1.09 (0.67)	1.25 (0.64)	1.39 (1.22)	1.59 (0.89)	1.91 (1.23)	7.79x10^-27^
Systolic blood pressure [mmHg]	119.94 (19.09)	121.52 (16.38)	125.01 (18.58)	123.09 (17.78)	126.97 (18.53)	3.22x10^-5^
Diastolic blood pressure [mmHg]	75.14 (10.25)	75.81 (8.89)	76.77 (9.97)	75.84 (9.78)	77.5 (10.32)	0.083
CD8^+^ T cells +#	0.08 (0.05)	0.09 (0.06)	0.10 (0.07)	0.11 (0.07)	0.12 (0.07)	3.46x10^-13^
CD4^+^ T cells +	0.16 (0.06)	0.17 (0.06)	0.16 (0.06)	0.17 (0.06)	0.16 (0.06)	1
Natural killer cells +#	0.02 (0.02)	0.02 (0.02)	0.03 (0.03)	0.03 (0.03)	0.03 (0.03)	0.060
B cells +#	0.05 (0.04)	0.05 (0.03)	0.05 (0.02)	0.05 (0.02)	0.05 (0.02)	1
Monocytes +	0.11 (0.02)	0.11 (0.02)	0.12 (0.02)	0.12 (0.02)	0.12 (0.03)	9.40x10^-11^
Granulocytes +	0.65 (0.09)	0.63 (0.09)	0.63 (0.09)	0.62 (0.09)	0.62 (0.09)	9.87x10^-6^
**Categorical phenotypes**	**number**	**number (p-value)**	**number (p-value)**	**number (p-value)**	**number (p-value)**	**-**
Sex [male/female]	83/207	122/167 (1.50x10^-3^*)	127/163 (1.00x10^-3^ *)	156/133 (5.00x10^-4^ *)	194/96 (5.00x10^-4^ *)	-
Glucose status [combination of IFG and IGT/IFG/IGT/NGT]	3/8/21/258	8/6/46/229 (3.50x10^-3^ *)	11/13/40/226 (4.00x10^-3^ *)	6/23/46/214 (5.00x10^-4^ *)	21/22/55/192 (5.00x10^-4^ *)	-

Means, standard deviations and p-values for trend are presented for the different quintiles for the continuous phenotypes. For the categorical variables total numbers of individuals in the different quintiles and p-values for the comparison of the corresponding quintile vs quintile 1 are given.

# variables were log transformed for determination of p-values.

* p-values are still significant after Bonferroni adjustment. + Proportions of cell types were estimated using method developed by Houseman *et al*. [32]

^1^ Variable only available for 1,440 samples, distribution between the quintiles (288/288/288/288/288).

^2^ Variable only available for 617 samples, distribution between the quintiles (124/123/123/123/124). IFG: impaired fasting glucose. IGT: impaired glucose tolerance. NGT: normal glucose tolerance.

### Associations between methylation at the 18 identified significant CpG sites and transcript levels within a ± 500kb cis region

The investigations of the associations between DNA methylation and gene expression at nearby genes (± 500kb cis area around significant CpG sites) were based on a subset of 533 participants with gene expression data available (S3 Table). Analyses were performed using linear regression and B-H correction for multiple testing. The methylation level at the CpG site cg06500161 (*ABCG1*) showed an inverse association with the *ABCG1* gene expression level (B-H-adjusted p-value = 1.5x10^-9^) after adjustment for age, sex, BMI, smoking, estimated white blood cell proportions, technical variables, and plate (Table 5). This association was also detectable after additional adjustment for fasting glucose, 2-hour glucose, fasting insulin, 2-hour insulin or HOMA-IR (B-H-adjusted p-values = 1.2x10^-8^, 1.0x10^-8^, 2.3x10^-9^, 6.2x10^-8^, 3.3x10^-9^, respectively). The associations of gene expression levels with the other 30 significant CpG sites that were identified in the methylation analysis described above were not significant (S14 Table).

**Table 5:** Summary of the analysis for association between DNA methylation and gene expression, and between DNA methylation and phenotypes.

	Coefficient	p-value	B-H-adj. p-value
**Association of methylation at cg06500161 (*ABCG1*) with different phenotypes**			
**fasting glucose**	0.044	4.2x10^-10^	9.2x10^-5^
**2-hour glucose**	0.042	4.6x10^-9^	2.0x10^-3^
**fasting insulin**	0.043	6.9x10^-9^	3.0x10^-3^
**2-hour insulin**	0.058	2.9x10^-7^	9.0x10^-6^
**HOMA-IR**	0.047	3.9x10^-10^	1.7x10^-4^
**Association of methylation at cg06500161 and gene expression level of a transcript annotated to *ABCG1* (ILMN_2329927)**			
**no adj. for phenotype**	-3.647	1.9x10^-12^	1.5x10^-9^
**adj. for fasting glucose**	-3.515	1.6x10^-11^	1.2x10^-8^
**adj. for 2-hour glucose**	-3.535	1.3x10^-11^	1.0x10^-8^
**adj. for fasting insulin**	-3.640	3.0x10^-12^	2.3x10^-9^
**adj. for 2-hour insulin**	-3.419	8.0x10^-11^	6.2x10^-8^
**adj. for HOMA-IR**	-3.617	4.3x10^-12^	3.3x10^-9^

The coefficient and unadjusted as well as B-H-adjusted p-value are presented.

### Ingenuity Pathway Analysis

Pathway analyses based on the 1,000 CpG sites with the lowest p-values for association with fasting glucose showed an enrichment of fasting glucose-associated CpG sites (in model 1 and 2) in the pathway “Ephrin Receptor Signaling” at B-H-corrected levels of significance (p-values 0.041 and 0.035) (Table 6 and Table 7). Furthermore, “Phospholipase C Signaling” showed suggestive evidence for enrichment in model 1 (B-H-adjusted p-value = 0.090) and in model 2 (B-H-adjusted p-values = 0.103) (Table 6 and Table 7). Also “Axonal Guidance Signaling”, “Adipogenesis Pathway”, and “Molecular Mechanisms of Cancer” were detectable in both adjustment models (B-H-adjusted p-values 0.110 and 0.057) (Table 6 and Table 7). Furthermore, “ERK/MAPK Signaling” pathway shows enrichment for fasting glucose-associated CpG sites in model 1 (B-H-adjusted p value = 0.110) (Table 6). Finally, “Leptin Signaling in Obesity” shows enrichment for genes detected within the top 1,000 for adjustment model 2 as well “Ephrin A Signaling” (B-H-adjusted p-values = 0.057). Results for the top ten canonical pathways for 2-hour glucose, fasting insulin, and HOMA-IR are shown in S15, S16, S17, S18, S19 and S20 Tables.

**Table 6:** Pathway analysis based on the top 1,000 CpG sites associated with fasting glucose (for results from model 1).

Ingenuity Canonical Pathways	B-H-adj. p-value	Ratio
Ephrin Receptor Signaling	0.041	17/172
Phospholipase C Signaling	0.090	19/231
Axonal Guidance Signaling	0.110	28/427
Molecular Mechanisms of Cancer	0.110	24/359
CCR3 Signaling in Eosinophils	0.110	11/113
ERK/MAPK Signaling	0.110	15/186
Adipogenesis pathway	0.110	12/132
Aldosterone Signaling in Epithelial Cells	0.110	13/151
Renal Cell Carcinoma Signaling	0.110	8/69
Role of NFAT in Cardiac Hypertrophy	0.110	14/176

The p-value corrected using the Benjamini-Hochberg method for multiple testing and the ratio of the number of genes uploaded in the software/total number of genes included in the pathway are presented for each pathway. Underlined pathways are significant after correction for multiple testing using Benjamini-Hochberg.

**Table 7:** Pathway analysis based on the top 1,000 CpG sites associated with fasting glucose (for results from model 2).

Ingenuity Canonical Pathways	B-H-adj. p-value	Ratio
Ephrin Receptor Signaling	0.035	17/172
Axonal Guidance Signaling	0.057	29/427
Adipogenesis pathway	0.057	13/132
Molecular Mechanisms of Cancer	0.057	25/359
Leptin Signaling in Obesity	0.057	9/73
RhoGDI Signaling	0.057	15/172
AMPK Signaling	0.057	15/177
Ephrin A Signaling	0.057	7/48
PPARα/RXRα Activation	0.072	14/165
Phospholipase C Signaling	0.103	17/231

The p-value corrected using the Benjamini-Hochberg method for multiple testing and the ratio of the number of genes uploaded in the software/total number of genes included in the pathway are presented for each pathway. Underlined pathways are significant after correction for multiple testing using Benjamini-Hochberg.

## Discussion

To the best of our knowledge associations between methylation patterns in whole blood samples and parameters of glucose metabolism have not been previously reported in a large population-based study on a genome-wide scale. In the present study, we identified 31 CpG sites related to fasting glucose, 2-hour glucose, fasting insulin, 2-hour insulin, or HOMA-IR, independent of age, sex, smoking, and estimated white blood cell proportions. Three of these were also significantly associated with fasting insulin and seven with 2-hour insulin after additional adjustment for BMI (model 2). Additionally methylation of three CpG sites was significantly associated with fasting insulin in model 2. Furthermore, we found suggestive evidence for associations of methylation with 2-hour insulin and HOMA-IR after adjustment for BMI. By comparing the coefficients of each CpG site across the models, we determined the reduction of effect size through adjustment for BMI to be around 30% of the association. The general function of the detected genes is summarized in S21 Table. In an additional analysis, we found the strongest association between DNA methylation and different phenotypes and estimated white blood cell proportion for cg06500161. But also for all other CpG sites (excluded cg13016916), which were significantly associated with our markers of glucose metabolism after BMI adjustment, we detected associations with different phenotypes, mainly for estimated white blood cell proportions. As a second step we demonstrated an inverse association between DNA methylation and gene expression of the *ABCG1* gene. By performing an enrichment analysis of CpG sites for known biological pathways we detected several pathways which can be linked to diabetes. One aim of epigenetic studies is the elucidation of missing heritability for type 2 diabetes, related traits and many other outcomes. In this context, it is important to note that our findings cannot directly contribute to the clarification of missing heritability due to the cross-sectional study design. In order to address this aspect, prospective studies based on families will be required for which both genetic and epigenetic data are available so that their respective contributions to the heritable component of diabetes or glycaemic traits can be estimated.

### Involvement of *ABCG1* in type 2 diabetes

Using whole blood, we found association between the DNA methylation level of a CpG site annotated to *ABCG1* (ATP-binding cassette, sub-family G (WHITE), member 1) and all investigated phenotypes relating to glucose metabolism except HbA1c. Furthermore, DNA methylation of this CpG site was also inversely associated with its expression. Our findings support previous evidence by Hidalgo *et al*. who found an association between cg06500161 (*ABCG1*) and fasting insulin, as well as HOMA-IR, in the Genetics of Lipid Lowering Drugs and Diet Network (GOLDN) study in CD4^+^ T cells. The authors did not adjust for BMI. In our study associations of cg06500161 with fasting glucose and 2-hour glucose (the latter not analyzed in their study) could be detected, and we further identified additional CpG sites associated with T2D-related traits that were not identified in the GOLDN study [35]. Possible explanations for the partly different findings include the different DNA sources (whole blood in our study versus CD4^+^ T cells in theirs), differences in data normalization, differences in multiple testing correction and different sample sizes. In our study, the associations with fasting glucose, 2-hour glucose, and HOMA-IR were attenuated when adjusting for BMI. As ABCG1 is an important regulator of cholesterol efflux from macrophages to HDL (high density lipoprotein) [36], this might have been expected considering that altered lipid levels, obesity and diabetes are associated, as shown in an analysis of serum samples from more than 5,000 men and women participating in the Framingham Heart Study [37]. Furthermore, low HDL-cholesterol is known as an independent risk factor for T2D [38]. Also in another study using our KORA samples an association between DNA methylation and lipid levels could be observed [39]. However, the connection between *ABCG1* and T2D/related traits as well as dyslipidemia is further supported by animal and human studies [40–45]. Taken together, our findings support a role of the *ABCG1* gene in the regulation of glucose metabolism and suggest that epigenetic mechanisms are involved in the association.

It is known that DNA methylation can influence the expression of genes [46–49]. From our findings that DNA methylation and gene expression of *ABCG1* are associated independently of our investigated phenotypes, we conclude that DNA methylation and gene expression may affect each other and therefore may have an influence on insulin or glucose levels. Our results cannot provide evidence for the direction of influence.

### Relevance of the other associated CpG sites for type 2 diabetes

*SREBF1* (sterol regulatory element binding transcription factor 1), for which a significant association between methylation status and fasting glucose and 2-hour insulin was observed, has been linked to diabetes or related traits in human studies before [50, 51]. Also *TNF* (tumor necrosis factor) has been implicated in the development of type 2 diabetes. This latter gene encodes the proinflammatory cytokine TNFalpha that is secreted mainly by macrophages and overexpressed in adipose tissue [52]. It is involved in the regulation of lipid metabolism and insulin resistance [53, 54]. Animal studies demonstrate an association between TNFalpha and diabetes [55–57]. In cell culture experiments an association between TNFalpha and insulin resistance was observed [58, 59]. In human studies it was shown that the level of this cytokine is increased in T2D patients and subjects with insulin resistance [60, 61]. For *CPT1A* (carnitine palmitoyltransferase 1a), which is a key enzyme of fatty acid transport into mitochondria for beta-oxidation [62, 63], a link to diabetes or related traits can be found in animal and expression studies [63–65]. However, when analyzing SNPs at the *CPT1A* locus in diabetic and non-diabetic individuals, no association with T2D, hepatic lipid content or insulin resistance in T2D was observed [66]. Furthermore, a link between *PALLD* (palladin, cytoskeletal associated protein) and obesity has been reported [67]. For the amino acid transporter *SLC1A5* an enhanced expression was observed in goat mammary gland epithelial transfected cells [68]. Furthermore, the overexpression of GLUT1 and GLUT12 increases the expression of *SLC1A5* in goat mammary gland epithelial transfected cells [69]. Diaz *et al*. conclude from their experiments using transgenic mice overexpressing chicken Ski that *Ski* plays a main role in skeletal muscle metabolism and adipogenesis and therefore influence risk of obesity and diabetes [70]. This result was supported by findings of Leong *et al*. [71]. For *PXN* it was shown that glucose induces the activation of this paxillin, which is mediated by beta 1 integrin intracellular signaling [72].

### Involvement of BMI in DNA methylation association

We showed that effect strengths were reduced on average by around 30% after adjustment for BMI, suggesting an influence of BMI on the investigated phenotypes.

In the literature evidence can be found that BMI is associated with DNA methylation patterns. For example Na *et al*. showed a differential influence of BMI on global DNA methylation when analyzing healthy women. They found a U-shaped association between BMI and Alu methylation, where the lowest methylation degree was found at a BMI between 23 and 30 kg/m^2^. These findings imply an involvement of BMI-related changes in Alu methylation in the etiology and pathogenesis of obesity [73]. Furthermore, allele-specific, age-dependent, and BMI-associated methylation at *MCHR1* was shown in human blood samples [74]. Furthermore, we could demonstrate in our study by trend analysis that the degree of methylation at cg06500161 rose with increasing measures of BMI. However, functional and/or time-series studies are needed to elucidate potential cause and effect mechanisms in the association between these genes, adiposity and glucose mechanisms.

As the previously discussed findings of our study indicate that BMI influences methylation, it is not unexpected that adjustment for BMI diminishes the association of selected CpG sites with the investigated measurements of glucose metabolism. Some CpG sites where associations seem to be mediated by BMI are biologically plausible in the context of T2D or related traits.

### Association of gene expression and DNA methylation

We demonstrate an inverse association between DNA methylation and gene expression of the *ABCG1* gene. In general this finding is not surprising due to the fact that hypermethylation within the gene promotor and shores is mostly associated with a reduced gene expression [75–78]. Recent studies demonstrate that methylation in the gene body may lead to increased rather than decreased gene expression [79, 80], which would be in contrast to our findings. However, due to lack of general evidence further studies will be required to elucidate the mechanisms underlying our findings, as this cannot be achieved in an epidemiological setting. The particularly strong association between methylation at cg06500161 and the markers of glucose metabolism seems reflected by the fact that we can only demonstrate an association of methylation and gene expression for this gene after correction for multiple testing, whereas associations for other CpG sites were significant only before correction for multiple testing. It should be noted that the sample size for the gene expression analyses was limited to 533 samples and therefore statistical power may have been lower compared to the analysis between methylation and phenotypes. Additional 23 CpG sites show an association with gene expression in the unadjusted analysis (S14 Table).

### Enrichment of signals from biological pathways and link to diabetes

In order to functionally integrate our results, we conducted a pathway analysis based on the 1,000 top hits for each phenotype and each model. Some of the identified pathways have a well-established link to diabetes or related traits. One example is the “Adipogenesis Pathway”. An *et al*. show that cyclin Y is involved in adipogenesis and lipid accumulation and that its inhibition could be a therapeutic approach to obesity and diabetes [81]. Furthermore, it was shown that adipose cell expansion is associated with abdominal obesity and insulin resistance [82] and that there is an association between genetic predisposition for T2D and adipocyte hypertrophy [83]. Another example is “Phospholipase C Signaling”. A contribution of phospholipase C delta1 to obesity through regulation of thermogenesis and adipogenesis in mice was observed [84], as were its effects on insulin secretion in a pancreatic beta-cell line [85]. A link to diabetes can also be found for “Leptin Signaling in Obesity”, as leptin is involved in the regulation of obesity, which in turn is an important risk factor for T2D [86]. A further link to diabetes exists for “Ephrin Receptor Signaling” and “Ephrin A/B Signaling”. It was shown that EphA and ephrin A regulate insulin secretion [87] and that the communication between ephrin receptors and ephrin in exocrine and endocrine cells is involved in pancreatic function [88]. Ephrin receptors and ephrins are expressed in pancreatic beta-cells in humans and mice [89] and EphAs are tyrosine phosphorylated under low glucose concentrations and initiate forward signaling, which in turn reduces insulin secretion [90]. Finally, there is evidence for linking “ERK/MAPK Signaling” to diabetes, as it was shown that MAPK/ERK signaling controls glucose metabolism by regulating insulin sensitivity in Drosophila [91]. Furthermore, an association between sustained activation of ERK signaling in adipocytes and the pathogenesis of T2D was found [92]. Additional, Hsp60 activates ERK1/2 in skeletal muscle cells and thus inhibits insulin signaling and insulin-stimulated glucose uptake [93]. Taken together, we observe an enrichment of our detected genes in pathways which are connected to diabetes or related traits. From this we conclude that our results, although not always significant, are biologically plausible in the context of measures of glucose metabolism and may be the basis for further analysis.

### Strengths/Limitations

The main strength of this study is the use of whole blood samples, as whole blood is readily available in the clinical routine, unlike tissue biopsies, and therefore has greater relevance in the context of the prediction of diabetes. On the other hand, this is also a limitation, as it is known that DNA methylation patterns differ across tissues [32] and whole blood thus represents a mixture of cell types and may differ in methylation profile from insulin-responsive tissues like liver, skeletal muscle and adipose tissue. However, we adjusted for estimated white blood cell proportions derived from methylation data for blood cells in order to reduce confounding due to inter-individual differences in blood cell proportions.

Further strengths of our study are the genome-wide approach and the population-based design. Our study also included oral glucose tolerance tests so that data for 2-hour glucose and 2-hour insulin were available. However, a major caveat of our cross-sectional study design is that it does not allow deductions on cause and effect. Finally, meta-analyses of EWAS of glycemic traits will have a larger power to detect further CpG sites whose methylation levels did not reach statistical significance in our study.

## Conclusion

Our study presents evidence of association between DNA methylation and measures of glucose metabolism, using whole blood samples from individuals with European ancestry. Our findings show that epigenetic markers associated with measures of glucose metabolism can be detected in whole blood samples, and they confirm previous evidence that *ABCG1* is involved in diabetes either directly or indirectly. Furthermore, we confirm recent findings found in CD4^+^ T cells–as well as from participants with Indian Asian ancestry–from the GOLDN and LOLIPOP studies, which demonstrated an association between methylation degree of cg06500161 (*ABCG1*) and measures of glucose metabolism and incident T2D [35, 45]. In addition we detected enrichment of differentially methylated genes in pathways which are biologically plausible in the context of diabetes. The findings provide evidence that DNA methylation may be associated with T2D and related traits, a relationship which can be measured in DNA isolated from whole blood. Particularly, epigenetic patterns of disease-relevant tissues might further advance our understanding of how DNA methylation at the identified genes is involved in diabetes and therefore its pathophysiology. However, for ethical and practical reasons, DNA methylation analyses are often only feasible in whole blood rather than in disease-relevant tissues, particularly in large population-based observational studies.

## Supporting Information

S1 Table Characteristics of the study population (n = 1,440) for the DNA methylation analyses of fasting insulin and HOMA-IR. (DOC)

S2 Table Characteristics of the study population (n = 617) for the DNA methylation analyses of 2-hour insulin. (DOC)

S3 Table Characteristics of the study population (n = 533) for the gene expression analyses. (DOC)

S4 Table Associations between DNA methylation at cg09694782 (unannotated) and different phenotypes, based on quintiles of methylation level. Means, standard deviations and p-values for trend are presented for the different quintiles for the continuous phenotypes. For the categorical variables total numbers of individuals in the different quintiles and p-values for the comparison of the corresponding quintile vs the quintile 1 are given.
(DOC)

S5 Table Associations between DNA methylation at cg13016916 (*CREB3L2*) and different phenotypes, based on quintiles of methylation level. Means, standard deviations and p-values for trend are presented for the different quintiles for the continuous phenotypes. For the categorical variables total numbers of individuals in the different quintiles and p-values for the comparison of the corresponding quintile vs the quintile 1 are given.
(DOC)

S6 Table Associations between DNA methylation at cg11990813 (*KIAA0664*) and different phenotypes, based on quintiles of methylation level. Means, standard deviations and p-values for trend are presented for the different quintiles for the continuous phenotypes. For the categorical variables total numbers of individuals in the different quintiles and p-values for the comparison of the corresponding quintile vs the quintile 1 are given.
(DOC)

S7 Table Associations between DNA methylation at cg11307565 (*PXN*) and different phenotypes, based on quintiles of methylation level. Means, standard deviations and p-values for trend are presented for the different quintiles for the continuous phenotypes. For the categorical variables total numbers of individuals in the different quintiles and p-values for the comparison of the corresponding quintile vs the quintile 1 are given.
(DOC)

S8 Table Associations between DNA methylation at cg06946797 (unannotated) and different phenotypes, based on quintiles of methylation level. Means, standard deviations and p-values for trend are presented for the different quintiles for the continuous phenotypes. For the categorical variables total numbers of individuals in the different quintiles and p-values for the comparison of the corresponding quintile vs the quintile 1 are given.
(DOC)

S9 Table Associations between DNA methylation at cg03979241 (*EPB49*) and different phenotypes, based on quintiles of methylation level. Means, standard deviations and p-values for trend are presented for the different quintiles for the continuous phenotypes. For the categorical variables total numbers of individuals in the different quintiles and p-values for the comparison of the corresponding quintile vs the quintile 1 are given.
(DOC)

S10 Table Associations between DNA methylation at cg23899654 (unannotated) and different phenotypes, based on quintiles of methylation level. Means, standard deviations and p-values for trend are presented for the different quintiles for the continuous phenotypes. For the categorical variables total numbers of individuals in the different quintiles and p-values for the comparison of the corresponding quintile vs the quintile 1 are given.
(DOC)

S11 Table Associations between DNA methylation at cg22065733 (unannotated) and different phenotypes, based on quintiles of methylation level. Means, standard deviations and p-values for trend are presented for the different quintiles for the continuous phenotypes. For the categorical variables total numbers of individuals in the different quintiles and p-values for the comparison of the corresponding quintile vs the quintile 1 are given.
(DOC)

S12 Table Associations between DNA methylation at cg17266233 (*DGKZ*) and different phenotypes, based on quintiles of methylation level. Means, standard deviations and p-values for trend are presented for the different quintiles for the continuous phenotypes. For the categorical variables total numbers of individuals in the different quintiles and p-values for the comparison of the corresponding quintile vs the quintile 1 are given.
(DOC)

S13 Table Associations between DNA methylation at cg03581271 (*PALLD*) and different phenotypes, based on quintiles of methylation level. Means, standard deviations and p-values for trend are presented for the different quintiles for the continuous phenotypes. For the categorical variables total numbers of individuals in the different quintiles and p-values for the comparison of the corresponding quintile vs the quintile 1 are given.
(DOC)

S14 Table Summary of the analysis for association between DNA methylation and gene expression showing the top association per CpG sites for unadjusted p-values < 0.05. (DOC)

S15 Table Pathway analysis based on the top 1,000 CpG sites associated with 2-hour glucose (for results from model 1). The table gives p-values corrected using the Benjamini-Hochberg method for multiple testing and the ratio of the number of genes uploaded in the software/total number of genes included in the pathway are presented for each pathway. Underlined pathways are significant after correction for multiple testing using Benjamini-Hochberg.
(DOC)

S16 Table Pathway analysis based on the top 1,000 CpG sites associated with 2-hour glucose (for results from model 2). The table gives p-values corrected using the Benjamini-Hochberg method for multiple testing and the ratio of the number of genes uploaded in the software/total number of genes included in the pathway are presented for each pathway. Underlined pathways are significant after correction for multiple testing using Benjamini-Hochberg.
(DOC)

S17 Table Pathway analysis based on the top 1,000 CpG sites associated with fasting insulin (for results from model 1). The table gives p-values corrected using the Benjamini-Hochberg method for multiple testing and the ratio of the number of genes uploaded in the software/total number of genes included in the pathway are presented for each pathway.
(DOC)

S18 Table Pathway analysis based on the top 1,000 CpG sites associated with fasting insulin (for results from model 2). The table gives p-values corrected using the Benjamini-Hochberg method for multiple testing and the ratio of the number of genes uploaded in the software/total number of genes included in the pathway are presented for each pathway. Underlined pathways are significant after correction for multiple testing using Benjamini-Hochberg.
(DOC)

S19 Table Pathway analysis based on the top 1,000 CpG sites associated with HOMA-IR (for results from model 1). The table gives p-values corrected using the Benjamini-Hochberg method for multiple testing and the ratio of the number of genes uploaded in the software/total number of genes included in the pathway are presented for each pathway.
(DOC)

S20 Table Pathway analysis based on the top 1,000 CpG sites associated with HOMA-IR (for results from model 2). The table gives p-values corrected using the Benjamini-Hochberg method for multiple testing and the ratio of the number of genes uploaded in the software/total number of genes included in the pathway are presented for each pathway. Underlined pathways are significant after correction for multiple testing using Benjamini-Hochberg.
(DOC)

S21 Table Function of genes showing significant associations between methylation status and parameters of glucose metabolism. Informations were taken from www.ncbi.nlm.nih.gov/gene/ in February 2016, if not stated otherwise.
(DOC)
