# Supplementary material for: Association between DNA Methylation in Whole Blood and Measures of Glucose Metabolism: KORA F4 Study
Source: PLoS One. 2016 Mar 28;11(3):e0152314. doi: 10.1371/journal.pone.0152314 (PMC4809492; doi:10.1371/journal.pone.0152314)
Supplement: S1 Table — (DOC) [file pone.0152314.s001.doc]

**S1 Table.** **Characteristics of the study population (n=1,440) for the DNA methylation analyses of fasting insulin and HOMA-IR.**

|  | **Median**  **(25^th^; 75^th^ percentile)** | **%** |
| --- | --- | --- |
| **Sex [% male]** | - | 47.2 |
| **Age [years]** | 59 (53; 67) | - |
| **BMI [kg/m^2^]** | 27.1 (24.5; 30.1) | - |
| **Waist circumference [cm]** | 93.6 (84.5; 102.3) | - |
|  |  |  |
| **Fasting serum glucose [mmol/l]** | 5.3 (4.9; 5.6) | - |
| **2-hour serum glucose [mmol/l]** | 6.0 (5.0; 7.2) | - |
| **HbA1c [%]** | 5.5 (5.2; 5.7) | - |
| **Glucose tolerance status [%]**  NGT  IFG  IGT  Combined IFG and IGT | -  -  -  - | 77.3  4.9  14.4  3.4 |
| **Fasting insulin [µlU/ml]** | 4.1 (2.8; 6.7) | - |
| **2-hour Insulin [µlU/ml]** | 50.2 (28.7; 78.1) | - |
| **HOMA-IR** | 1.0 (0.6; 1.6) | - |
|  |  |  |
| **C-reactive protein [mg/l]** | 1.1 (0.6; 2.2) | - |
| **Leucocytes [/nl]** | 5.5 (4.7; 6.5) | - |
| **Cholesterol [mmol/l]** | 5.7 (5.1; 6.4) | - |
| **Triglycerides [mmol/l]** | 1.2 (0.9; 1.7) | - |
| **Systolic blood pressure [mmHg]** | 122.2 (111.0; 134.5) | - |
| **Diastolic blood pressure [mmHg]** | 75.5 (69.5; 82.5) | - |
|  |  |  |
| **Alcohol consumption [g/day]** | 8.6 (0.0; 22.9) | - |
| **Smoking status [%]**  never  ex  current | -  -  - | 44.9  40.0  15.1 |
| **Physically active [%]**  (combination of activity during summer and winter with >= 1 hour per week) | - | 60.1 |

NGT: normal glucose tolerance

IFG: impaired fasting glucose

IGT: impaired glucose tolerance
