# Supplementary material for: Association between DNA Methylation in Whole Blood and Measures of Glucose Metabolism: KORA F4 Study
Source: PLoS One. 2016 Mar 28;11(3):e0152314. doi: 10.1371/journal.pone.0152314 (PMC4809492; doi:10.1371/journal.pone.0152314)
Supplement: S2 Table — (DOC) [file pone.0152314.s002.doc]

**S2 Table.** **Characteristics of the study population (n=617) for the DNA methylation analyses of 2-hour insulin**.

|  | **Median**  **(25^th^; 75^th^ percentile)** | **%** |
| --- | --- | --- |
| **Sex [% male]** | - | 48.3 |
| **Age [years]** | 68 (65; 72) | - |
| **BMI [kg/m^2^]** | 27.6 (25.2; 30.3) | - |
| **Waist circumference [cm]** | 96.1 (88.4; 103.5) | - |
|  |  |  |
| **Fasting serum glucose [mmol/l]** | 5.4 (5.1; 5.7) | - |
| **2-hour serum glucose [mmol/l]** | 6.5 (5.4; 7.8) | - |
| **HbA1c [%]** | 5.6 (5.3; 5.8) | - |
| **Glucose tolerance status [%]**  NGT  IFG  IGT  Combined IFG and IGT | -  -  -  - | 68.6  5.7  20.3  5.5 |
| **Fasting insulin [µlU/ml]** | 4.7 (3.3; 7.5) | - |
| **2-hour insulin [µlU/ml]** | 50.2 (28.7; 78.1) | - |
| **HOMA-IR** | 1.1 (0.8; 1.8) | - |
|  |  |  |
| **C-reactive protein [mg/l]** | 1.4 (0.7; 2.5) | - |
| **Leucocytes [/nl]** | 5.6 (4.8; 6.4) | - |
| **Cholesterol [mmol/l]** | 5.8 (5.1; 6.5) | - |
| **Triglycerides [mmol/l]** | 1.3 (0.9; 1.7) | - |
| **Systolic blood pressure [mmHg]** | 126.5 (114.5; 138.0) | - |
| **Diastolic blood pressure [mmHg]** | 74.5 (68.5; 81.5) | - |
|  |  |  |
| **Alcohol consumption [g/day]** | 7.6 (0.0; 20.0) | - |
| **Smoking status [%]**  never  ex  current | -  -  - | 52.8  39.2  8.0 |
| **Physically active [%]**  (combination of activity during summer and winter with >= 1 hour per week) | - | 56.9 |

NGT: normal glucose tolerance

IFG: impaired fasting glucose

IGT: impaired glucose tolerance
