# Supplementary material for: Association between DNA Methylation in Whole Blood and Measures of Glucose Metabolism: KORA F4 Study
Source: PLoS One. 2016 Mar 28;11(3):e0152314. doi: 10.1371/journal.pone.0152314 (PMC4809492; doi:10.1371/journal.pone.0152314)
Supplement: S3 Table — (DOC) [file pone.0152314.s003.doc]

**S3 Table. Characteristics of the study population (n=533) for the gene expression analyses.**

|  | **Median**  **(25^th^; 75^th^ percentile)** | **%** |
| --- | --- | --- |
| **Sex [% male]** | - | 47.7 |
| **Age [years]** | 68 (65; 72) | - |
| **BMI [kg/m^2^]** | 27.8 (25.2; 30.5) | - |
| **Waist circumference [cm]** | 95.8 (88.6; 103.7) | - |
|  |  |  |
| **Fasting serum glucose [mmol/l]** | 5.4 (5.1; 5.7) | - |
| **2-hour serum glucose [mmol/l]** | 6.4 (5.4; 7.7) | - |
| **HbA1c [%]** | 5.5 (5.3; 5.7) | - |
| **Glucose tolerance status [%]**  NGT  IFG  IGT  Combined IFG and IGT | -  -  -  - | 70.2  5.4  18.6  5.8 |
| **Insulin [µlU/ml]** | 4.7 (3.3; 7.4) | - |
| **2-hour insulin [µlU/ml]** | 49.8 (28.5; 77.3) | - |
| **HOMA-IR** | 1.1 (0.8; 1.8) | - |
|  |  |  |
| **C-reactive protein [mg/l]** | 1.3 (0.7; 2.5) | - |
| **Leucocytes [/nl]** | 5.6 (4.7; 6.4) | - |
| **Cholesterol [mmol/l]** | 5.8 (5.1; 6.5) | - |
| **Triglycerides [mmol/l]** | 1.2 (0.9; 1.7) | - |
| **Systolic blood pressure [mmHg]** | 126.5 (114.5; 138.5) | - |
| **Diastolic blood pressure [mmHg]** | 74.5 (68.5; 81.5) | - |
|  |  |  |
| **Alcohol consumption [g/day]** | 7.9 (0.0; 20.4) | - |
| **Smoking status [%]**  never  ex  current | -  -  - | 53.3  39.8  6.9 |
| **Physically active [%]**  (combination of activity during summer and winter with >= 1 hour per week) | - | 55.9 |

NGT: normal glucose tolerance

IFG: impaired fasting glucose

IGT: impaired glucose tolerance
