# Supplementary material for: Association between DNA Methylation in Whole Blood and Measures of Glucose Metabolism: KORA F4 Study
Source: PLoS One. 2016 Mar 28;11(3):e0152314. doi: 10.1371/journal.pone.0152314 (PMC4809492; doi:10.1371/journal.pone.0152314)
Supplement: S4 Table — Means, standard deviations and p-values for trend are presented for the different quintiles for the continuous phenotypes. For the categorical variables total numbers of individuals in the different quintiles and p-values for the comparison of the corresponding quintile vs the quintile 1 are given. (DOC) [file pone.0152314.s004.doc]

**S4 Table. Associations between DNA methylation at cg09694782 (unannotated) and different phenotypes, based on quintiles of methylation level.**

|  | **Quintile 1**  **(n=290)** | **Quintile 2**  **(n=289)** | **Quintile 3**  **(n=289)** | **Quintile 4**  **(n=289)** | **Quintile 5**  **(n=290)** |  |
| --- | --- | --- | --- | --- | --- | --- |
| **Continuous phenotype** | **Mean (SD)** | **Mean (SD)** | **Mean (SD)** | **Mean (SD)** | **Mean (SD)** | **p for trend (Bonf. adjusted)** |
| Age [years] # | 61.29 (8.6) | 59.54 (8.65) | 59.69 (8.66) | 59.81 (9.13) | 58.92 (8.47) | 0.032 |
| BMI [kg/m^2^] # | 27.59 (4.49) | 27.93 (4.6) | 27.5 (4.41) | 27.53 (4.26) | 27.04 (3.96) | 0.672 |
| Waist circumference [cm] | 94.21 (13.29) | 94.38 (13.4) | 93.92 (13.32) | 92.75 (12.07) | 92.88 (12.34) | 0.799 |
| Fasting glucose [mmol/l] # | 5.38 (0.55) | 5.28 (0.5) | 5.35 (0.53) | 5.24 (0.49) | 5.29 (0.55) | 0.221 |
| 2-hour glucose [mmol/l] # | 6.45 (1.77) | 6.17 (1.73) | 6.3 (1.72) | 6.03 (1.63) | 6.14 (1.67) | 0.190 |
| HbA1c [%] | 5.48 (0.32) | 5.47 (0.35) | 5.49 (0.32) | 5.45 (0.31) | 5.46 (0.3) | 1 |
| C-reactive protein [mg/l] # | 1.89 (1.88) | 1.66 (1.52) | 1.69 (1.54) | 1.83 (1.73) | 1.56 (1.61) | 0.822 |
| Fasting insulin [µlU/ml] # ^1^ | 7.08 (6.95) | 6.66 (6.82) | 6.60 (7.49) | 5.77 (6.42) | 5.15 (5.54) | 1.41x10^-3^ |
| 2-hour insulin [µlU/ml] # ^2^ | 69.78 (55.85) | 63.99 (48.58) | 63.56 (44.18) | 57.45 (44.72) | 57.01 (57.34) | 0.289 |
| HOMA-IR # ^1^ | 1.74 (1.83) | 1.60 (1.72) | 1.63 (1.99) | 1.38 (1.61) | 1.26 (1.53) | 2.46x10^-3^ |
| Cholesterol [mmol/l] # | 5.77 (1.08) | 5.83 (1.03) | 5.84 (1.01) | 5.79 (0.98) | 5.76 (0.91) | 1 |
| Triglycerides [mmol/l] # | 1.38 (0.81) | 1.49 (0.92) | 1.53 (1.29) | 1.4 (0.97) | 1.43 (0.97) | 1 |
| Systolic blood pressure [mm Hg] | 123.21 (20.53) | 123.27 (17.43) | 124.1 (18.22) | 122.17 (18.17) | 123.74 (16.73) | 1 |
| Diastolic blood pressure [mm Hg] | 76.14 (10.78) | 76.5 (9.41) | 76.67 (9.94) | 75.4 (9.59) | 76.38 (9.61) | 1 |
| CD8^+^ T cells # | 0.11 (0.07) | 0.1 (0.07) | 0.1 (0.07) | 0.1 (0.07) | 0.11 (0.07) | 2.14x10^-73^ |
| CD4^+^ T cells | 0.16 (0.06) | 0.16 (0.06) | 0.16 (0.06) | 0.16 (0.06) | 0.16 (0.06) | 1.95x10^-11^ |
| Natural killer cells # | 0.02 (0.02) | 0.02 (0.02) | 0.03 (0.03) | 0.02 (0.02) | 0.02 (0.02) | 1.13x10^-8^ |
| B cells # | 0.05 (0.03) | 0.05 (0.02) | 0.05 (0.02) | 0.05 (0.04) | 0.05 (0.02) | 1.17x10^-5^ |
| Monocytes | 0.12 (0.02) | 0.12 (0.03) | 0.12 (0.02) | 0.12 (0.03) | 0.12 (0.03) | 9.65x10^-7^ |
| Granulocytes | 0.63 (0.08) | 0.63 (0.09) | 0.64 (0.09) | 0.64 (0.1) | 0.63 (0.09) | 1.27x10^-11^ |
| **Categorial phenotypes** | **number** | **number (p-value)** | **number (p-value)** | **number (p-value)** | **number (p-value)** | **-** |
| Sex [male/female] | 147/143 | 128/161 (0.120) | 141/148 (0.625) | 119/170 (0.023) | 146/144 (0.936) | - |
| Glucose status [combination of IFG and IGT/IFG/IGT/NGT] | 14/21/52/203 | 8/13/39/229 (0.079) | 12/12/47/218 (0.365) | 5/10/31/243 (1.0x10^-3^ *) | 10/16/39/225 (0.230) | - |

Means, standard deviations and p-values for trend are presented for the different quintiles for the continuous phenotypes. For the categorical variables total numbers of individuals in the different quintiles and p-values for the comparison of the corresponding quintile vs the quintile 1 are given.

# variables were log transformed for determination of p-values

* p-values are still significant after Bonferroni adjustment

+ Proportions of cell types were estimated using method developed by Houseman *et al.* (1)

^1^ Variable only available for 1,440 samples; distribution between the quintiles (288/288/287/288/288)

^2^ Variable only available for 617 samples, distribution between the quintiles (123/124/122/124/123)

IFG: impaired fasting glucose

IGT: impaired glucose tolerance

NGT: normal glucose tolerance

**Reference**

1. Houseman EA, Accomando WP, Koestler DC, Christensen BC, Marsit CJ, Nelson HH, et al. DNA methylation arrays as surrogate measures of cell mixture distribution. BMC Bioinformatics. 2012;13:86.
