# Supplementary material for: Association between DNA Methylation in Whole Blood and Measures of Glucose Metabolism: KORA F4 Study
Source: PLoS One. 2016 Mar 28;11(3):e0152314. doi: 10.1371/journal.pone.0152314 (PMC4809492; doi:10.1371/journal.pone.0152314)
Supplement: S5 Table — Means, standard deviations and p-values for trend are presented for the different quintiles for the continuous phenotypes. For the categorical variables total numbers of individuals in the different quintiles and p-values for the comparison of the corresponding quintile vs the quintile 1 are given. (DOC) [file pone.0152314.s005.doc]

**S5 Table. Associations between DNA methylation at cg13016916 (*CREB3L2*) and different phenotypes, based on quintiles of methylation level.**

|  | **Quintile 1**  **(n=286)** | **Quintile 2**  **(n=286)** | **Quintile 3**  **(n=286)** | **Quintile 4**  **(n=286)** | **Quintile 5**  **(n=286)** |  |
| --- | --- | --- | --- | --- | --- | --- |
| **Continuous phenotype** | **Mean (SD)** | **Mean (SD)** | **Mean (SD)** | **Mean (SD)** | **Mean (SD)** | **p for trend (Bonf. adjusted)** |
| Age [years] # | 59.59 (8.77) | 60.08 (8.64) | 60.13 (8.67) | 59.34 (8.57) | 60.29 (9.02) | 1 |
| BMI [kg/m^2^] # | 27.46 (4.19) | 27.66 (4.4) | 27.3 (4.31) | 27.51 (4.47) | 27.52 (4.39) | 1 |
| Waist circumference [cm] | 93.47 (12.53) | 94.51 (13.35) | 93.11 (12.6) | 93.64 (13.76) | 93.3 (12.4) | 1 |
| Fasting glucose [mmol/l] # | 5.28 (0.54) | 5.29 (0.54) | 5.32 (0.53) | 5.35 (0.51) | 5.32 (0.51) | 1 |
| 2-hour glucose [mmol/l] # | 6.09 (1.63) | 6.29 (1.77) | 6.14 (1.65) | 6.34 (1.84) | 6.23 (1.65) | 1 |
| HbA1c [%] | 5.47 (0.31) | 5.47 (0.33) | 5.44 (0.31) | 5.48 (0.34) | 5.48 (0.32) | 1 |
| C-reactive protein [mg/l] # | 1.78 (1.74) | 1.77 (1.6) | 1.56 (1.62) | 1.72 (1.69) | 1.79 (1.7) | 1 |
| Fasting insulin [µlU/ml] # ^1^ | 5.20 (5.66) | 6.22 (6.28) | 6.04 (6.27) | 7.21 (8.15) | 6.52 (6.77) | 0.058 |
| 2-hour insulin [µlU/ml] # ^2^ | 61.08 (56.41) | 55.67 (37.93) | 62.50 (46.14) | 64.06 (53.40) | 69.50 (56.22) | 1 |
| HOMA-IR # ^1^ | 1.26 (1.57) | 1.51 (1.61) | 1.48 (1.65) | 1.75 (2.04) | 1.59 (1.82) | 0.084 |
| Cholesterol [mmol/l] # | 5.78 (0.98) | 5.85 (1.08) | 5.82 (1.05) | 5.74 (0.94) | 5.8 (0.98) | 1 |
| Triglycerides [mmol/l] # | 1.4 (0.87) | 1.49 (1.01) | 1.5 (1.33) | 1.43 (0.9) | 1.4 (0.85) | 1 |
| Systolic blood pressure [mm Hg] | 123.96 (17.7) | 122.83 (18) | 122.88 (17.71) | 123.43 (18.1) | 123.65 (19.75) | 1 |
| Diastolic blood pressure [mm Hg] | 76.28 (9.83) | 76.06 (10.06) | 75.93 (9.43) | 76.74 (9.86) | 76.15 (10.28) | 1 |
| CD8^+^ T cells # | 0.1 (0.07) | 0.1 (0.07) | 0.1 (0.06) | 0.1 (0.06) | 0.1 (0.07) | 1 |
| CD4^+^ T cells | 0.16 (0.06) | 0.16 (0.06) | 0.17 (0.06) | 0.17 (0.06) | 0.17 (0.06) | 0.911 |
| Natural killer cells # | 0.03 (0.03) | 0.03 (0.03) | 0.02 (0.02) | 0.03 (0.03) | 0.02 (0.02) | 0.291 |
| B cells # | 0.05 (0.04) | 0.05 (0.02) | 0.05 (0.02) | 0.05 (0.02) | 0.05 (0.02) | 0.362 |
| Monocytes | 0.12 (0.02) | 0.12 (0.02) | 0.12 (0.02) | 0.12 (0.02) | 0.12 (0.03) | 1 |
| Granulocytes | 0.63 (0.1) | 0.64 (0.08) | 0.63 (0.09) | 0.62 (0.08) | 0.63 (0.08) | 1 |
| **Categorial phenotypes** | **number** | **number (p-value)** | **number (p-value)** | **number (p-value)** | **number (p-value)** | **-** |
| sex [male/female] | 138/148 | 140/146 (0.712) | 131/155 (0.805) | 139/147 (0.744) | 129/157 (0.664) | - |
| glucose status [combination of IFG and IGT/IFG/IGT/NGT] | 8/12/35/231 | 11/17/43/215 (0.355) | 10/19/40/217 (0.416) | 9/12/50/215 (0.252) | 11/11/39/225 (0.748) | - |

Means, standard deviations and p-values for trend are presented for the different quintiles for the continuous phenotypes. For the categorical variables total numbers of individuals in the different quintiles and p-values for the comparison of the corresponding quintile vs the quintile 1 are given.

# variables were log transformed for determination of p-values

* p-values are still significant after Bonferroni adjustment

+ Proportions of cell types were estimated using method developed by Houseman *et al.* (1)

^1^ Variable only available for 1,440 samples, distribution between the quintiles (285/284/284/284/285)

^2^ Variable only available for 617 samples, distribution between the quintiles (123/122/123/122/123)

IFG: impaired fasting glucose

IGT: impaired glucose tolerance

NGT, normal glucose tolerance

**Reference**

1. Houseman EA, Accomando WP, Koestler DC, Christensen BC, Marsit CJ, Nelson HH, et al. DNA methylation arrays as surrogate measures of cell mixture distribution. BMC Bioinformatics. 2012;13:86.
