# Supplementary material for: Association between DNA Methylation in Whole Blood and Measures of Glucose Metabolism: KORA F4 Study
Source: PLoS One. 2016 Mar 28;11(3):e0152314. doi: 10.1371/journal.pone.0152314 (PMC4809492; doi:10.1371/journal.pone.0152314)
Supplement: S6 Table — Means, standard deviations and p-values for trend are presented for the different quintiles for the continuous phenotypes. For the categorical variables total numbers of individuals in the different quintiles and p-values for the comparison of the corresponding quintile vs the quintile 1 are given. (DOC) [file pone.0152314.s006.doc]

**S6 Table. Associations between DNA methylation at cg11990813 (*KIAA0664*) and different phenotypes, based on quintiles of methylation level.**

|  | **Quintile 1**  **(n=290)** | **Quintile 2**  **(n=289)** | **Quintile 3**  **(n=290)** | **Quintile 4**  **(n=289)** | **Quintile 5**  **(n=290)** |  |
| --- | --- | --- | --- | --- | --- | --- |
| **Continuous phenotype** | **Mean (SD)** | **Mean (SD)** | **Mean (SD)** | **Mean (SD)** | **Mean (SD)** | **p for trend (Bonf. adjusted)** |
| Age [years] # | 60.11 (8.32) | 59.93 (8.71) | 59.91 (8.87) | 59.63 (8.92) | 59.70 (8.86) | 1 |
| BMI [kg/m^2^] # | 27.18 (4.01) | 27.19 (4.37) | 27.75 (4.43) | 27.50 (4.38) | 27.99 (4.55) | 0.191 |
| Waist circumference [cm] | 92.53 (12.65) | 93.30 (13.05) | 94.27 (12.93) | 93.24 (12.85) | 94.90 (13.06) | 0.474 |
| Fasting glucose [mmol/l] # | 5.31 (0.57) | 5.28 (0.50) | 5.36 (0.52) | 5.27 (0.52) | 5.32 (0.52) | 1 |
| 2-hour glucose [mmol/l] # | 6.17 (1.73) | 6.17 (1.67) | 6.24 (1.73) | 6.16 (1.76) | 6.34 (1.66) | 1 |
| HbA1c [%] | 5.49 (0.3) | 5.44 (0.31) | 5.49 (0.33) | 5.46 (0.33) | 5.46 (0.32) | 1 |
| C-reactive protein [mg/l] # | 1.60 (1.63) | 1.73 (1.64) | 1.64 (1.54) | 1.83 (1.77) | 1.83 (1.72) | 0.748 |
| Fasting insulin [µlU/ml] # ^1^ | 5.63 (6.51) | 5.75 (6.26) | 6.35 (6.68) | 6.12 (6.21) | 7.44 (7.65) | 0.016 |
| 2-hour insulin [µlU/ml] # ^2^ | 55.04 (45.97) | 61.45 (58.81) | 63.93 (50.02) | 64.70 (44.51) | 66.86 (51.75) | 0.594 |
| HOMA-IR # ^1^ | 1.37 (1.68) | 1.40 (1.69) | 1.56 (1.76) | 1.48 (1.60) | 1.81 (1.97) | 0.036 |
| Cholesterol [mmol/l] # | 5.78 (0.97) | 5.81 (1.08) | 5.76 (0.95) | 5.81 (1.00) | 5.82 (1.02) | 1 |
| Triglycerides [mmol/l] # | 1.43 (0.86) | 1.49 (1.35) | 1.44 (0.90) | 1.43 (0.92) | 1.45 (0.91) | 1 |
| Systolic blood pressure [mm Hg] | 122.49 (18.17) | 125.28 (19.1) | 122.78 (16.89) | 122.08 (18.46) | 123.9 (18.48) | 1 |
| Diastolic blood pressure [mm Hg] | 76.05 (10.02) | 77.00 (10.05) | 75.38 (9.73) | 75.83 (10.17) | 76.81 (9.36) | 1 |
| CD8^+^ T cells # | 0.12 (0.07) | 0.11 (0.07) | 0.10 (0.06) | 0.10 (0.06) | 0.09 (0.06) | 1.33x10^-7^ |
| CD4^+^ T cells | 0.16 (0.06) | 0.16 (0.06) | 0.17 (0.06) | 0.17 (0.06) | 0.17 (0.06) | 0.014 |
| Natural killer cells # | 0.02 (0.03) | 0.02 (0.02) | 0.03 (0.03) | 0.03 (0.03) | 0.03 (0.03) | 0.010 |
| B cells # | 0.05 (0.04) | 0.05 (0.02) | 0.05 (0.02) | 0.05 (0.02) | 0.05 (0.02) | 1 |
| Monocytes | 0.12 (0.02) | 0.12 (0.03) | 0.12 (0.02) | 0.12 (0.02) | 0.12 (0.03) | 1 |
| Granulocytes | 0.61 (0.09) | 0.63 (0.09) | 0.63 (0.08) | 0.63 (0.09) | 0.64 (0.08) | 1.58x10^-3^ |
| **Categorial phenotypes** | **number** | **number (p-value)** | **number (p-value)** | **number (p-value)** | **number (p-value)** | **-** |
| sex [male/female] | 138/152 | 146/143 (0.518) | 137/153 (1.000) | 124/165 (0.277) | 137/153 (1.000) | - |
| glucose status [combination of IFG and IGT/IFG/IGT/NGT] | 12/20/35/223 | 7/13/38/231 (0.388) | 10/19/46/215 (0.617) | 10/10/36/233 (0.290) | 10/10/53/217 (0.062) | - |

Means, standard deviations and p-values for trend are presented for the different quintiles for the continuous phenotypes. For the categorical variables total numbers of individuals in the different quintiles and p-values for the comparison of the corresponding quintile vs the quintile 1 are given.

# variables were log transformed for determination of p-values

* p-values are still significant after Bonferroni adjustment

+ Proportions of cell types were estimated using method developed by Houseman *et al.* (1)

^1^ Variable only available for 1,440 samples, distribution between the quintiles (288/288/288/288/288)

^2^ Variable only available for 617 samples, distribution between the quintiles (124/123/123/123/124)

IFG: impaired fasting glucose

IGT: impaired glucose tolerance

NGT, normal glucose tolerance

**Reference**

1. Houseman EA, Accomando WP, Koestler DC, Christensen BC, Marsit CJ, Nelson HH, et al. DNA methylation arrays as surrogate measures of cell mixture distribution. BMC Bioinformatics. 2012;13:86.
