# Supplementary material for: Association between DNA Methylation in Whole Blood and Measures of Glucose Metabolism: KORA F4 Study
Source: PLoS One. 2016 Mar 28;11(3):e0152314. doi: 10.1371/journal.pone.0152314 (PMC4809492; doi:10.1371/journal.pone.0152314)
Supplement: S7 Table — Means, standard deviations and p-values for trend are presented for the different quintiles for the continuous phenotypes. For the categorical variables total numbers of individuals in the different quintiles and p-values for the comparison of the corresponding quintile vs the quintile 1 are given. (DOC) [file pone.0152314.s007.doc]

**S7 Table. Associations between DNA methylation at cg11307565 (*PXN*) and different phenotypes, based on quintiles of methylation level.**

|  | **Quintile 1**  **(n=290)** | **Quintile 2**  **(n=289)** | **Quintile 3**  **(n=290)** | **Quintile 4**  **(n=289)** | **Quintile 5**  **(n=290)** |  |
| --- | --- | --- | --- | --- | --- | --- |
| **Continuous phenotype** | **Mean (SD)** | **Mean (SD)** | **Mean (SD)** | **Mean (SD)** | **Mean (SD)** | **p for trend (Bonf. adjusted)** |
| Age [years] # | 60.91 (8.32) | 59.11 (8.70) | 59.79 (8.81) | 59.99 (8.81) | 59.49 (8.94) | 1 |
| BMI [kg/m^2^] # | 27.74 (4.26) | 27.91 (4.45) | 27.86 (4.56) | 27.23 (4.14) | 26.88 (4.29) | 0.037 |
| Waist circumference [cm] | 94.14 (12.29) | 94.66 (13.02) | 94.73 (13.63) | 92.34 (12.59) | 92.38 (12.88) | 0.201 |
| Fasting glucose [mmol/l] # | 5.40 (0.55) | 5.32 (0.53) | 5.28 (0.52) | 5.27 (0.53) | 5.27 (0.50) | 0.021 |
| 2-hour glucose [mmol/l] # | 6.27 (1.69) | 6.30 (1.67) | 6.29 (1.70) | 6.25 (1.81) | 5.97 (1.67) | 0.396 |
| HbA1c [%] | 5.48 (0.32) | 5.48 (0.32) | 5.47 (0.32) | 5.47 (0.30) | 5.45 (0.33) | 1 |
| C-reactive protein [mg/l] # | 1.72 (1.62) | 1.67 (1.64) | 1.68 (1.61) | 1.85 (1.72) | 1.71 (1.73) | 1 |
| Fasting insulin [µlU/ml] # ^1^ | 6.79 (7.18) | 6.66 (7.78) | 6.70 (6.77) | 5.91 (5.37) | 5.24 (6.07) | 0.021 |
| 2-hour insulin [µlU/ml] # ^2^ | 65.97 (55.56) | 62.06 (47.74) | 65.75 (48.33) | 66.23 (51.90) | 52.00 (47.62) | 0.828 |
| HOMA-IR # ^1^ | 1.70 (2.00) | 1.62 (1.97) | 1.61 (1.74) | 1.42 (1.35) | 1.27 (1.58) | 0.014 |
| Cholesterol [mmol/l] # | 5.91 (1.08) | 5.87 (1.02) | 5.73 (1.01) | 5.79 (0.96) | 5.70 (0.94) | 0.082 |
| Triglycerides [mmol/l] # | 1.51 (0.95) | 1.46 (0.86) | 1.43 (0.83) | 1.43 (1.29) | 1.41 (1.03) | 1 |
| Systolic blood pressure [mm Hg] | 124.00 (18.56) | 123.94 (19.25) | 124.04 (18.14) | 120.60 (17.06) | 123.94 (18.01) | 1 |
| Diastolic blood pressure [mm Hg] | 76.26 (9.73) | 77.10 (11.00) | 76.58 (9.45) | 74.84 (9.52) | 76.29 (9.51) | 1 |
| CD8^+^ T cells # | 0.12 (0.08) | 0.10 (0.06) | 0.10 (0.07) | 0.09 (0.06) | 0.08 (0.05) | 2.28x10^-13^ |
| CD4^+^ T cells | 0.18 (0.07) | 0.18 (0.06) | 0.17 (0.06) | 0.16 (0.06) | 0.14 (0.05) | 4.22x10^-17^ |
| Natural killer cells # | 0.03 (0.03) | 0.03 (0.03) | 0.02 (0.02) | 0.02 (0.02) | 0.02 (0.02) | 2.31x10^-6^ |
| B cells # | 0.05 (0.03) | 0.05 (0.02) | 0.05 (0.02) | 0.05 (0.02) | 0.05 (0.03) | 0.485 |
| Monocytes | 0.12 (0.02) | 0.12 (0.02) | 0.12 (0.02) | 0.11 (0.03) | 0.12 (0.03) | 1 |
| Granulocytes | 0.58 (0.08) | 0.61 (0.08) | 0.63 (0.08) | 0.65 (0.08) | 0.68 (0.08) | 1.98x10^-57^ |
| **Categorial phenotypes** | **number** | **number (p-value)** | **number (p-value)** | **number (p-value)** | **number (p-value)** | **-** |
| sex [male/female] | 136/154 | 145/144 (0.455) | 134/156 (0.942) | 123/166 (0.311) | 144/146 (0.553) | - |
| glucose status [combination of IFG and IGT/IFG/IGT/NGT] | 13/20/41/216 | 11/16/45/217 (0.863) | 7/12/44/227 (0.247) | 11/10/45/223 (0.288) | 7/14/33/236 (0.215) | - |

Means, standard deviations and p-values for trend are presented for the different quintiles for the continuous phenotypes. For the categorical variables total numbers of individuals in the different quintiles and p-values for the comparison of the corresponding quintile vs the quintile 1 are given.

# variables were log transformed for determination of p-values

* p-values are still significant after Bonferroni adjustment

+ Proportions of cell types were estimated using method developed by Houseman *et al.* (1)

^1^ Variable only available for 1,440 samples, distribution between the quintiles (288/288/288/288/288)

^2^ Variable only available for 617 samples, distribution between the quintiles (124/123/123/123/124)

IFG: impaired fasting glucose

IGT: impaired glucose tolerance

NGT, normal glucose tolerance

**Reference**

1. Houseman EA, Accomando WP, Koestler DC, Christensen BC, Marsit CJ, Nelson HH, et al. DNA methylation arrays as surrogate measures of cell mixture distribution. BMC Bioinformatics. 2012;13:86.
