# Supplementary material for: Association between DNA Methylation in Whole Blood and Measures of Glucose Metabolism: KORA F4 Study
Source: PLoS One. 2016 Mar 28;11(3):e0152314. doi: 10.1371/journal.pone.0152314 (PMC4809492; doi:10.1371/journal.pone.0152314)
Supplement: S8 Table — Means, standard deviations and p-values for trend are presented for the different quintiles for the continuous phenotypes. For the categorical variables total numbers of individuals in the different quintiles and p-values for the comparison of the corresponding quintile vs the quintile 1 are given. (DOC) [file pone.0152314.s008.doc]

**S8 Table. Associations between DNA methylation at cg06946797 (unannotated) and different phenotypes, based on quintiles of methylation level.**

|  | **Quintile 1**  **(n=289)** | **Quintile 2**  **(n=288)** | **Quintile 3**  **(n=288)** | **Quintile 4**  **(n=288)** | **Quintile 5**  **(n=289)** |  |
| --- | --- | --- | --- | --- | --- | --- |
| **Continuous phenotype** | **Mean (SD)** | **Mean (SD)** | **Mean (SD)** | **Mean (SD)** | **Mean (SD)** | **p for trend (Bonf. adjusted)** |
| Age [years] # | 60.98 (9.14) | 61.00 (8.92) | 59.67 (8.27) | 59.29 (8.40) | 58.33 (8.61) | 1.71x10^-4^ |
| BMI [kg/m^2^] # | 27.92 (4.52) | 28.35 (4.69) | 27.47 (4.31) | 27.01 (4.12) | 26.80 (3.93) | 7.47x10^-5^ |
| Waist circumference [cm] | 95.18 (12.99) | 95.25 (13.01) | 93.44 (12.61) | 92.89 (12.85) | 91.38 (12.78) | 2.65x10^-4^ |
| Fasting glucose [mmol/l] # | 5.35 (0.53) | 5.30 (0.51) | 5.33 (0.55) | 5.33 (0.52) | 5.23 (0.51) | 0.177 |
| 2-hour glucose [mmol/l] # | 6.40 (1.81) | 6.24 (1.64) | 6.21 (1.71) | 6.19 (1.66) | 6.07 (1.72) | 0.332 |
| HbA1c [%] | 5.47 (0.32) | 5.49 (0.34) | 5.49 (0.30) | 5.45 (0.32) | 5.44 (0.31) | 0.861 |
| C-reactive protein [mg/l] # | 2.09 (1.80) | 1.99 (1.81) | 1.68 (1.56) | 1.56 (1.60) | 1.30 (1.38) | 6.66x10^-10^ |
| Fasting insulin [µlU/ml] # ^1^ | 7.13 (7.28) | 6.74 (6.97) | 6.10 (6.98) | 5.92 (5.68) | 5.42 (6.44) | 8.69x10^-3^ |
| 2-hour insulin [µlU/ml] # ^2^ | 73.24 (58.07) | 67.52 (54.65) | 60.90 (52.14) | 54.00 (39.35) | 56.18 (43.93) | 0.015 |
| HOMA-IR # ^1^ | 1.75 (1.94) | 1.65 (1.83) | 1.48 (1.81) | 1.45 (1.50) | 1.29 (1.63) | 6.06x10^-3^ |
| Cholesterol [mmol/l] # | 5.80 (1.09) | 5.74 (1.01) | 5.84 (1.03) | 5.73 (0.95) | 5.89 (0.93) | 1 |
| Triglycerides [mmol/l] # | 1.49 (1.36) | 1.43 (0.89) | 1.42 (0.88) | 1.40 (0.81) | 1.47 (0.94) | 1 |
| Systolic blood pressure [mm Hg] | 121.76 (18.08) | 122.90 (17.54) | 124.51 (19.04) | 124.01 (18.74) | 123.35 (17.86) | 1 |
| Diastolic blood pressure [mm Hg] | 75.00 (10.24) | 75.71 (9.19) | 76.90 (10.25) | 77.07 (10.14) | 76.47 (9.45) | 0.225 |
| CD8^+^ T cells # | 0.10 (0.08) | 0.11 (0.07) | 0.11 (0.07) | 0.10 (0.06) | 0.09 (0.05) | 0.012 |
| CD4^+^ T cells | 0.15 (0.06) | 0.16 (0.06) | 0.16 (0.06) | 0.17 (0.06) | 0.18 (0.06) | 7.13x10^-12^ |
| Natural killer cells # | 0.02 (0.02) | 0.02 (0.02) | 0.03 (0.03) | 0.03 (0.02) | 0.04 (0.03) | 1.78x10^-17^ |
| B cells # | 0.05 (0.04) | 0.05 (0.03) | 0.05 (0.02) | 0.05 (0.02) | 0.05 (0.02) | 1 |
| Monocytes | 0.12 (0.03) | 0.12 (0.02) | 0.12 (0.03) | 0.12 (0.02) | 0.11 (0.02) | 1 |
| Granulocytes | 0.64 (0.09) | 0.64 (0.09) | 0.63 (0.09) | 0.62 (0.08) | 0.62 (0.09) | 2.78x10^-3^ |
| **Categorial phenotypes** | **number** | **number (p-value)** | **number (p-value)** | **number (p-value)** | **number (p-value)** | **-** |
| sex [male/female] | 136/153 | 122/166 (0.366) | 147/141 (0.276) | 149/139 (0.229) | 127/162 (0.575) | - |
| glucose status [combination of IFG and IGT/IFG/IGT/NGT] | 10/16/50/213 | 9/11/38/230 (0.397) | 13/20/44/211 (0.787) | 9/15/39/225 (0.672) | 7/10/37/235 (0.203) | - |

Means, standard deviations and p-values for trend are presented for the different quintiles for the continuous phenotypes. For the categorical variables total numbers of individuals in the different quintiles and p-values for the comparison of the corresponding quintile vs the quintile 1 are given.

# variables were log transformed for determination of p-values

* p-values are still significant after Bonferroni adjustment

+ Proportions of cell types were estimated using method developed by Houseman *et al.* (1)

^1^ Variable only available for 1,440 samples, distribution between the quintiles (287/287/286/287/287)

^2^ Variable only available for 617 samples, distribution between the quintiles (123/123/123/123/123)

IFG: impaired fasting glucose

IGT: impaired glucose tolerance

NGT, normal glucose tolerance

**Reference**

1. Houseman EA, Accomando WP, Koestler DC, Christensen BC, Marsit CJ, Nelson HH, et al. DNA methylation arrays as surrogate measures of cell mixture distribution. BMC Bioinformatics. 2012;13:86.
