# Supplementary material for: Association between DNA Methylation in Whole Blood and Measures of Glucose Metabolism: KORA F4 Study
Source: PLoS One. 2016 Mar 28;11(3):e0152314. doi: 10.1371/journal.pone.0152314 (PMC4809492; doi:10.1371/journal.pone.0152314)
Supplement: S9 Table — Means, standard deviations and p-values for trend are presented for the different quintiles for the continuous phenotypes. For the categorical variables total numbers of individuals in the different quintiles and p-values for the comparison of the corresponding quintile vs the quintile 1 are given. (DOC) [file pone.0152314.s009.doc]

**S9 Table. Associations between DNA methylation at cg03979241 (*EPB49*) and different phenotypes, based on quintiles of methylation level.**

|  | **Quintile 1**  **(n=290)** | **Quintile 2**  **(n=289)** | **Quintile 3**  **(n=290)** | **Quintile 4**  **(n=289)** | **Quintile 5**  **(n=290)** |  |
| --- | --- | --- | --- | --- | --- | --- |
| **Continuous phenotype** | **Mean (SD)** | **Mean (SD)** | **Mean (SD)** | **Mean (SD)** | **Mean (SD)** | **p for trend (Bonf. adjusted)** |
| Age [years] # | 61.27 (9.56) | 61.37 (8.41) | 58.84 (8.50) | 58.72 (8.44) | 59.08 (8.30) | 3.56x10^-4^ |
| BMI [kg/m^2^] # | 27.36 (4.18) | 27.42 (4.05) | 27.73 (4.44) | 27.38 (4.45) | 27.72 (4.67) | 1 |
| Waist circumference [cm] | 95.08 (12.70) | 93.80 (11.63) | 94.06 (13.05) | 92.81 (13.23) | 92.48 (13.81) | 0.101 |
| Fasting glucose [mmol/l] # | 5.32 (0.50) | 5.33 (0.52) | 5.29 (0.53) | 5.34 (0.54) | 5.25 (0.55) | 1 |
| 2-hour glucose [mmol/l] # | 6.25 (1.68) | 6.39 (1.80) | 6.18 (1.75) | 6.23 (1.63) | 6.03 (1.68) | 0.820 |
| HbA1c [%] | 5.45 (0.34) | 5.50 (0.29) | 5.46 (0.32) | 5.46 (0.33) | 5.47 (0.32) | 1 |
| C-reactive protein [mg/l] # | 1.80 (1.68) | 1.86 (1.78) | 1.74 (1.71) | 1.61 (1.55) | 1.63 (1.59) | 0.907 |
| Fasting insulin [µlU/ml] # ^1^ | 5.95 (7.17) | 5.97 (6.19) | 5.94 (5.75) | 6.55 (7.41) | 6.88 (6.84) | 0.616 |
| 2-hour insulin [µlU/ml] # ^2^ | 53.83 (53.44) | 65.03 (54.44) | 64.53 (47.68) | 64.41 (47.15) | 64.21 (49.02) | 1 |
| HOMA-IR # ^1^ | 1.46 (1.94) | 1.44 (1.55) | 1.44 (1.49) | 1.63 (2.03) | 1.64 (1.67) | 1 |
| Cholesterol [mmol/l] # | 5.67 (0.97) | 5.85 (0.95) | 5.78 (1.01) | 5.82 (1.08) | 5.87 (1.01) | 0.366 |
| Triglycerides [mmol/l] # | 1.36 (0.78) | 1.50 (1.33) | 1.46 (0.94) | 1.44 (0.93) | 1.47 (0.96) | 1 |
| Systolic blood pressure [mm Hg] | 125.93 (18.98) | 124.60 (17.24) | 121.81 (18.64) | 122.35 (17.46) | 121.84 (18.58) | 0.023 |
| Diastolic blood pressure [mm Hg] | 76.52 (10.71) | 75.99 (9.02) | 76.14 (9.84) | 76.47 (9.86) | 75.95 (9.92) | 1 |
| CD8^+^ T cells # | 0.06 (0.04) | 0.08 (0.05) | 0.10 (0.06) | 0.12 (0.07) | 0.14 (0.08) | 1.88x10^-62^ |
| CD4^+^ T cells | 0.13 (0.05) | 0.15 (0.05) | 0.16 (0.05) | 0.18 (0.06) | 0.20 (0.07) | 5.02x10^-52^ |
| Natural killer cells # | 0.03 (0.02) | 0.03 (0.02) | 0.03 (0.02) | 0.03 (0.03) | 0.03 (0.03) | 1 |
| B cells # | 0.04 (0.02) | 0.04 (0.02) | 0.05 (0.02) | 0.05 (0.02) | 0.06 (0.04) | 1.76x10^-35^ |
| Monocytes | 0.12 (0.02) | 0.12 (0.02) | 0.12 (0.02) | 0.12 (0.02) | 0.12 (0.03) | 1 |
| Granulocytes | 0.71 (0.07) | 0.66 (0.06) | 0.63 (0.06) | 0.60 (0.06) | 0.55 (0.08) | 1.01x10^-168^ |
| **Categorial phenotypes** | **number** | **number (p-value)** | **number (p-value)** | **number (p-value)** | **number (p-value)** | **-** |
| sex [male/female] | 188/102 | 134/155 (5.00x10^-4^ *) | 136/154 (5.00x10^-4^ *) | 127/162 (5.00x10^-4^ *) | 97/193 (5.00x10^-4^ *) | - |
| glucose status [combination of IFG and IGT/IFG/IGT/NGT] | 4/18/44/224 | 11/19/53/206 (0.184) | 12/9/39/230 (0.061) | 14/13/34/228 (0.056) | 8/13/38/231 (0.453) | - |

Means, standard deviations and p-values for trend are presented for the different quintiles for the continuous phenotypes. For the categorical variables total numbers of individuals in the different quintiles and p-values for the comparison of the corresponding quintile vs the quintile 1 are given.

# variables were log transformed for determination of p-values

* p-values are still significant after Bonferroni adjustment

+ Proportions of cell types were estimated using method developed by Houseman *et al.* (1)

^1^ Variable only available for 1,440 samples, distribution between the quintiles (288/288/288/288/288)

^2^ Variable only available for 617 samples, distribution between the quintiles (124/123/123/123/124)

IFG: impaired fasting glucose

IGT: impaired glucose tolerance

NGT, normal glucose tolerance

**Reference**

1. Houseman EA, Accomando WP, Koestler DC, Christensen BC, Marsit CJ, Nelson HH, et al. DNA methylation arrays as surrogate measures of cell mixture distribution. BMC Bioinformatics. 2012;13:86.
