# Supplementary material for: Association between DNA Methylation in Whole Blood and Measures of Glucose Metabolism: KORA F4 Study
Source: PLoS One. 2016 Mar 28;11(3):e0152314. doi: 10.1371/journal.pone.0152314 (PMC4809492; doi:10.1371/journal.pone.0152314)
Supplement: S10 Table — Means, standard deviations and p-values for trend are presented for the different quintiles for the continuous phenotypes. For the categorical variables total numbers of individuals in the different quintiles and p-values for the comparison of the corresponding quintile vs the quintile 1 are given. (DOC) [file pone.0152314.s010.doc]

**S10 Table. Associations between DNA methylation at cg23899654 (unannotated) and different phenotypes, based on quintiles of methylation level.**

|  | **Quintile 1**  **(n=290)** | **Quintile 2**  **(n=289)** | **Quintile 3**  **(n=290)** | **Quintile 4**  **(n=289)** | **Quintile 5**  **(n=290)** |  |
| --- | --- | --- | --- | --- | --- | --- |
| **Continuous phenotype** | **Mean (SD)** | **Mean (SD)** | **Mean (SD)** | **Mean (SD)** | **Mean (SD)** | **p for trend (Bonf. adjusted)** |
| Age [years] # | 59.88 (9.09) | 59.83 (8.92) | 59.36 (8.66) | 60.00 (8.57) | 60.22 (8.43) | 1 |
| BMI [kg/m^2^] # | 27.35 (4.41) | 27.61 (4.63) | 27.99 (4.34) | 27.41 (4.20) | 27.25 (4.19) | 1 |
| Waist circumference [cm] | 93.40 (13.13) | 93.28 (13.94) | 94.78 (12.75) | 94.06 (11.89) | 92.73 (12.79) | 1 |
| Fasting glucose [mmol/l] # | 5.27 (0.51) | 5.29 (0.52) | 5.35 (0.51) | 5.36 (0.55) | 5.28 (0.54) | 1 |
| 2-hour glucose [mmol/l] # | 6.01 (1.71) | 6.16 (1.66) | 6.22 (1.66) | 6.32 (1.75) | 6.38 (1.75) | 0.046 |
| HbA1c [%] | 5.47 (0.30) | 5.47 (0.33) | 5.49 (0.30) | 5.47 (0.33) | 5.46 (0.33) | 1 |
| C-reactive protein [mg/l] # | 1.65 (1.55) | 1.78 (1.76) | 1.80 (1.70) | 1.82 (1.79) | 1.59 (1.50) | 1 |
| Fasting insulin [µlU/ml] # ^1^ | 6.58 (7.40) | 6.62 (7.02) | 6.58 (6.80) | 5.41 (5.65) | 6.11 (6.49) | 1 |
| 2-hour insulin [µlU/ml] # ^2^ | 64.73 (60.21) | 65.26 (50.31) | 64.33 (45.65) | 57.85 (47.18) | 59.78 (47.92) | 1 |
| HOMA-IR # ^1^ | 1.61 (2.04) | 1.60 (1.79) | 1.59 (1.67) | 1.33 (1.54) | 1.48 (1.68) | 1 |
| Cholesterol [mmol/l] # | 5.74 (1.02) | 5.84 (0.98) | 5.78 (0.92) | 5.79 (1.03) | 5.83 (1.07) | 1 |
| Triglycerides [mmol/l] # | 1.43 (0.96) | 1.36 (0.82) | 1.52 (0.99) | 1.44 (1.30) | 1.47 (0.89) | 1 |
| Systolic blood pressure [mm Hg] | 122.21 (17.25) | 121.87 (19.27) | 124.51 (17.86) | 122.80 (18.54) | 125.14 (18.13) | 0.443 |
| Diastolic blood pressure [mm Hg] | 75.66 (9.02) | 75.27 (10.75) | 76.94 (9.97) | 76.46 (9.36) | 76.74 (10.15) | 0.937 |
| CD8^+^ T cells # | 0.09 (0.06) | 0.10 (0.07) | 0.10 (0.06) | 0.11 (0.07) | 0.11 (0.07) | 1.88x10^-62^ |
| CD4^+^ T cells | 0.17 (0.06) | 0.16 (0.06) | 0.17 (0.06) | 0.16 (0.06) | 0.17 (0.06) | 1 |
| Natural killer cells # | 0.03 (0.03) | 0.03 (0.03) | 0.03 (0.03) | 0.02 (0.02) | 0.02 (0.02) | 0.221 |
| B cells # | 0.05 (0.02) | 0.05 (0.02) | 0.05 (0.02) | 0.05 (0.04) | 0.05 (0.02) | 1 |
| Monocytes | 0.12 (0.02) | 0.12 (0.03) | 0.12 (0.02) | 0.12 (0.02) | 0.11 (0.02) | 1 |
| Granulocytes | 0.64 (0.08) | 0.64 (0.08) | 0.63 (0.09) | 0.63 (0.09) | 0.61 (0.09) | 1.92x10^-3^ |
| **Categorial phenotypes** | **number** | **number (p-value)** | **number (p-value)** | **number (p-value)** | **number (p-value)** | **-** |
| sex [male/female] | 131/159 | 130/159 (1.000) | 141/149 (0.473) | 151/138 (0.094) | 129/161 (0.931) | - |
| glucose status [combination of IFG and IGT/IFG/IGT/NGT] | 11/11/35/233 | 5/17/46/221 (0.155) | 6/19/44/221 (0.178) | 14/13/41/221 (0.710) | 13/12/42/223 (0.785) | - |

Means, standard deviations and p-values for trend are presented for the different quintiles for the continuous phenotypes. For the categorical variables total numbers of individuals in the different quintiles and p-values for the comparison of the corresponding quintile vs the quintile 1 are given.

# variables were log transformed for determination of p-values

* p-values are still significant after Bonferroni adjustment

+ Proportions of cell types were estimated using method developed by Houseman *et al.* (1)

^1^ Variable only available for 1,440 samples, distribution between the quintiles (288/288/288/288/288)

^2^ Variable only available for 617 samples, distribution between the quintiles (124/123/123/123/124)

IFG: impaired fasting glucose

IGT: impaired glucose tolerance

NGT, normal glucose tolerance

**Reference**

1. Houseman EA, Accomando WP, Koestler DC, Christensen BC, Marsit CJ, Nelson HH, et al. DNA methylation arrays as surrogate measures of cell mixture distribution. BMC Bioinformatics. 2012;13:86.
