# Supplementary material for: Association between DNA Methylation in Whole Blood and Measures of Glucose Metabolism: KORA F4 Study
Source: PLoS One. 2016 Mar 28;11(3):e0152314. doi: 10.1371/journal.pone.0152314 (PMC4809492; doi:10.1371/journal.pone.0152314)
Supplement: S11 Table — Means, standard deviations and p-values for trend are presented for the different quintiles for the continuous phenotypes. For the categorical variables total numbers of individuals in the different quintiles and p-values for the comparison of the corresponding quintile vs the quintile 1 are given. (DOC) [file pone.0152314.s011.doc]

**S11 Table. Associations between DNA methylation at cg22065733 (unannotated) and different phenotypes, based on quintiles of methylation level.**

|  | **Quintile 1**  **(n=289)** | **Quintile 2**  **(n=289)** | **Quintile 3**  **(n=289)** | **Quintile 4**  **(n=289)** | **Quintile 5**  **(n=290)** |  |
| --- | --- | --- | --- | --- | --- | --- |
| **Continuous phenotype** | **Mean (SD)** | **Mean (SD)** | **Mean (SD)** | **Mean (SD)** | **Mean (SD)** | **p for trend (Bonf. adjusted)** |
| Age [years] # | 59.84 (9.27) | 59.88 (8.20) | 59.68 (8.98) | 60.90 (8.35) | 59.03 (8.75) | 1 |
| BMI [kg/m^2^] # | 27.72 (4.54) | 27.90 (4.57) | 27.00 (4.13) | 27.87 (4.13) | 27.12 (4.36) | 1 |
| Waist circumference [cm] | 94.38 (13.13) | 94.41 (13.44) | 92.38 (12.78) | 94.56 (11.94) | 92.47 (13.16) | 1 |
| Fasting glucose [mmol/l] # | 5.29 (0.50) | 5.33 (0.53) | 5.35 (0.56) | 5.35 (0.51) | 5.24 (0.53) | 1 |
| 2-hour glucose [mmol/l] # | 6.15 (1.69) | 6.32 (1.79) | 6.22 (1.65) | 6.34 (1.79) | 6.07 (1.63) | 1 |
| HbA1c [%] | 5.45 (0.33) | 5.49 (0.32) | 5.46 (0.32) | 5.51 (0.32) | 5.44 (0.30) | 1 |
| C-reactive protein [mg/l] # | 1.82 (1.77) | 1.69 (1.64) | 1.70 (1.62) | 1.80 (1.68) | 1.63 (1.61) | 1 |
| Fasting insulin [µlU/ml] # ^1^ | 6.36 (6.72) | 7.13 (7.56) | 5.99 (6.66) | 6.32 (6.16) | 5.43 (6.23) | 0.257 |
| 2-hour insulin [µlU/ml] # ^2^ | 64.74 (48.47) | 63.56 (54.64) | 58.47 (43.36) | 68.36 (53.25) | 56.85 (51.73) | 1 |
| HOMA-IR # ^1^ | 1.54 (1.74) | 1.74 (1.98) | 1.46 (1.69) | 1.54 (1.58) | 1.32 (1.72) | 0.379 |
| Cholesterol [mmol/l] # | 5.72 (0.98) | 5.82 (0.95) | 5.84 (1.00) | 5.82 (1.06) | 5.80 (1.04) | 1 |
| Triglycerides [mmol/l] # | 1.41 (0.91) | 1.41 (0.86) | 1.54 (1.40) | 1.44 (0.82) | 1.43 (0.91) | 1 |
| Systolic blood pressure [mm Hg] | 123.65 (17.54) | 122.72 (18.15) | 122.44 (17.96) | 124.25 (19.01) | 123.53 (18.63) | 1 |
| Diastolic blood pressure [mm Hg] | 76.46 (9.39) | 75.86 (10.18) | 75.75 (9.78) | 76.89 (10.52) | 76.12 (9.53) | 1 |
| CD8^+^ T cells # | 0.09 (0.07) | 0.10 (0.06) | 0.10 (0.06) | 0.11 (0.06) | 0.11 (0.07) | 5.63x10^-4^ |
| CD4^+^ T cells | 0.17 (0.06) | 0.17 (0.06) | 0.17 (0.06) | 0.17 (0.06) | 0.16 (0.06) | 0.402 |
| Natural killer cells # | 0.02 (0.03) | 0.02 (0.02) | 0.03 (0.03) | 0.03 (0.02) | 0.03 (0.03) | 1 |
| B cells # | 0.05 (0.02) | 0.05 (0.02) | 0.05 (0.03) | 0.05 (0.02) | 0.05 (0.04) | 0.519 |
| Monocytes | 0.12 (0.02) | 0.12 (0.03) | 0.12 (0.02) | 0.12 (0.02) | 0.12 (0.02) | 1 |
| Granulocytes | 0.64 (0.08) | 0.64 (0.09) | 0.63 (0.09) | 0.62 (0.08) | 0.62 (0.09) | 0.011 |
| **Categorial phenotypes** | **number** | **number (p-value)** | **number (p-value)** | **number (p-value)** | **number (p-value)** | **-** |
| sex [male/female] | 139/150 | 134/155 (0.741) | 133/156 (0.699) | 132/157 (0.692) | 143/147 (0.795) | - |
| glucose status [combination of IFG and IGT/IFG/IGT/NGT] | 8/9/40/232 | 11/13/50/215 (0.359) | 15/20/33/221 (0.065) | 10/15/51/213 (0.203) | 5/15/34/236 (0.407) | - |

Means, standard deviations and p-values for trend are presented for the different quintiles for the continuous phenotypes. For the categorical variables total numbers of individuals in the different quintiles and p-values for the comparison of the corresponding quintile vs the quintile 1 are given.

# variables were log transformed for determination of p-values

* p-values are still significant after Bonferroni adjustment

+ Proportions of cell types were estimated using method developed by Houseman *et al.* (1)

^1^ Variable only available for 1,440 samples, distribution between the quintiles (288/287/288/287/288)

^2^ Variable only available for 617 samples, distribution between the quintiles (124/123/123/123/124)

IFG: impaired fasting glucose

IGT: impaired glucose tolerance

NGT, normal glucose tolerance

**Reference**

1. Houseman EA, Accomando WP, Koestler DC, Christensen BC, Marsit CJ, Nelson HH, et al. DNA methylation arrays as surrogate measures of cell mixture distribution. BMC Bioinformatics. 2012;13:86.
