# Supplementary material for: Association between DNA Methylation in Whole Blood and Measures of Glucose Metabolism: KORA F4 Study
Source: PLoS One. 2016 Mar 28;11(3):e0152314. doi: 10.1371/journal.pone.0152314 (PMC4809492; doi:10.1371/journal.pone.0152314)
Supplement: S12 Table — Means, standard deviations and p-values for trend are presented for the different quintiles for the continuous phenotypes. For the categorical variables total numbers of individuals in the different quintiles and p-values for the comparison of the corresponding quintile vs the quintile 1 are given. (DOC) [file pone.0152314.s012.doc]

**S12 Table. Associations between DNA methylation at cg17266233 (*DGKZ*) and different phenotypes, based on quintiles of methylation level.**

|  | **Quintile 1**  **(n=289)** | **Quintile 2**  **(n=288)** | **Quintile 3**  **(n=289)** | **Quintile 4**  **(n=288)** | **Quintile 5**  **(n=289)** |  |
| --- | --- | --- | --- | --- | --- | --- |
| **Continuous phenotype** | **Mean (SD)** | **Mean (SD)** | **Mean (SD)** | **Mean (SD)** | **Mean (SD)** | **p for trend (Bonf. adjusted)** |
| Age [years] # | 60.54 (9.09) | 60.19 (8.75) | 59.51 (8.79) | 59.51 (8.45) | 59.54 (8.55) | 1 |
| BMI [kg/m^2^] # | 27.79 (4.43) | 27.72 (4.27) | 27.58 (4.57) | 27.42 (4.38) | 27.15 (4.16) | 0.703 |
| Waist circumference [cm] | 94.31 (13.01) | 94.20 (12.16) | 93.41 (13.63) | 93.50 (13.14) | 92.85 (12.66) | 1 |
| Fasting glucose [mmol/l] # | 5.32 (0.55) | 5.31 (0.49) | 5.32 (0.53) | 5.32 (0.53) | 5.28 (0.53) | 1 |
| 2-hour glucose [mmol/l] # | 6.29 (1.73) | 6.31 (1.69) | 6.12 (1.75) | 6.21 (1.71) | 6.16 (1.67) | 1 |
| HbA1c [%] | 5.46 (0.32) | 5.45 (0.34) | 5.49 (0.31) | 5.46 (0.32) | 5.48 (0.30) | 1 |
| C-reactive protein [mg/l] # | 1.73 (1.67) | 1.89 (1.84) | 1.79 (1.75) | 1.70 (1.54) | 1.53 (1.50) | 0.850 |
| Fasting insulin [µlU/ml] # ^1^ | 6.86 (6.96) | 5.94 (6.05) | 6.65 (7.62) | 6.14 (6.5) | 5.72 (6.28) | 0.901 |
| 2-hour insulin [µlU/ml] # ^2^ | 68.54 (49.89) | 64.48 (54.32) | 53.40 (38.68) | 64.93 (61.01) | 60.93 (45.39) | 1 |
| HOMA-IR # ^1^ | 1.68 (1.85) | 1.44 (1.60) | 1.62 (2.00) | 1.49 (1.64) | 1.39 (1.63) | 0.964 |
| Cholesterol [mmol/l] # | 5.73 (1.01) | 5.79 (0.97) | 5.84 (1.03) | 5.86 (1.00) | 5.76 (1.00) | 1 |
| Triglycerides [mmol/l] # | 1.39 (0.92) | 1.48 (1.34) | 1.43 (0.88) | 1.45 (0.87) | 1.48 (0.95) | 1 |
| Systolic blood pressure [mm Hg] | 124.41 (18.57) | 123.08 (18.15) | 122.23 (18.00) | 123.44 (18.56) | 123.35 (18.03) | 1 |
| Diastolic blood pressure [mm Hg] | 76.56 (9.35) | 76.34 (10.09) | 75.57 (9.98) | 76.16 (9.91) | 76.45 (10.06) | 1 |
| CD8^+^ T cells # | 0.08 (0.06) | 0.10 (0.06) | 0.10 (0.07) | 0.11 (0.07) | 0.12 (0.06) | 7.39x10^-11^ |
| CD4^+^ T cells | 0.17 (0.06) | 0.16 (0.06) | 0.17 (0.06) | 0.17 (0.06) | 0.17 (0.06) | 1 |
| Natural killer cells # | 0.03 (0.03) | 0.02 (0.02) | 0.03 (0.02) | 0.03 (0.02) | 0.03 (0.03) | 1 |
| B cells # | 0.04 (0.02) | 0.05 (0.04) | 0.05 (0.03) | 0.05 (0.02) | 0.05 (0.02) | 4.55x10^-3^ |
| Monocytes | 0.12 (0.03) | 0.12 (0.03) | 0.11 (0.02) | 0.12 (0.02) | 0.12 (0.02) | 1 |
| Granulocytes | 0.66 (0.08) | 0.64 (0.09) | 0.63 (0.09) | 0.62 (0.08) | 0.60 (0.09) | 8.54x10^-15^ |
| **Categorial phenotypes** | **number** | **number (p-value)** | **number (p-value)** | **number (p-value)** | **number (p-value)** | **-** |
| sex [male/female] | 130/159 | 136/152 (0.754) | 127/162 (0.734) | 143/145 (0.355) | 142/147 (0.412) | - |
| glucose status [combination of IFG and IGT/IFG/IGT/NGT] | 11/16/49/213 | 7/14/45/222 (0.737) | 11/18/34/226 (0.364) | 12/13/43/220 (0.845) | 8/11/36/234 (0.252) | - |

Means, standard deviations and p-values for trend are presented for the different quintiles for the continuous phenotypes. For the categorical variables total numbers of individuals in the different quintiles and p-values for the comparison of the corresponding quintile vs the quintile 1 are given.

# variables were log transformed for determination of p-values

* p-values are still significant after Bonferroni adjustment

+ Proportions of cell types were estimated using method developed by Houseman *et al.* (1)

^1^ Variable only available for 1,440 samples, distribution between the quintiles (287/287/287/287/287)

^2^ Variable only available for 617 samples, distribution between the quintiles (123/123/122/123/123)

IFG: impaired fasting glucose

IGT: impaired glucose tolerance

NGT, normal glucose tolerance

**Reference**

1. Houseman EA, Accomando WP, Koestler DC, Christensen BC, Marsit CJ, Nelson HH, et al. DNA methylation arrays as surrogate measures of cell mixture distribution. BMC Bioinformatics. 2012;13:86.
