# Supplementary material for: Association between DNA Methylation in Whole Blood and Measures of Glucose Metabolism: KORA F4 Study
Source: PLoS One. 2016 Mar 28;11(3):e0152314. doi: 10.1371/journal.pone.0152314 (PMC4809492; doi:10.1371/journal.pone.0152314)
Supplement: S13 Table — Means, standard deviations and p-values for trend are presented for the different quintiles for the continuous phenotypes. For the categorical variables total numbers of individuals in the different quintiles and p-values for the comparison of the corresponding quintile vs the quintile 1 are given. (DOC) [file pone.0152314.s013.doc]

**S13 Table. Associations between DNA methylation at cg03581271 (*PALLD*) and different phenotypes, based on quintiles of methylation level.**

|  | **Quintile 1**  **(n=287)** | **Quintile 2**  **(n=287)** | **Quintile 3**  **(n=287)** | **Quintile 4**  **(n=287)** | **Quintile 5**  **(n=288)** |  |
| --- | --- | --- | --- | --- | --- | --- |
| **Continuous phenotype** | **Mean (SD)** | **Mean (SD)** | **Mean (SD)** | **Mean (SD)** | **Mean (SD)** | **p for trend (Bonf. adjusted)** |
| Age [years] # | 59.68 (8.98) | 60.57 (8.42) | 60.09 (8.55) | 59.14 (9.03) | 59.99 (8.65) | 1 |
| BMI [kg/m^2^] # | 27.70 (4.34) | 27.58 (4.08) | 27.78 (4.97) | 27.14 (4.07) | 27.41 (4.27) | 1 |
| Waist circumference [cm] | 94.09 (12.47) | 93.99 (12.11) | 93.77 (14.49) | 93.03 (12.39) | 93.31 (13.02) | 1 |
| Fasting glucose [mmol/l] # | 5.34 (0.56) | 5.30 (0.50) | 5.31 (0.53) | 5.29 (0.51) | 5.31 (0.53) | 1 |
| 2-hour glucose [mmol/l] # | 6.29 (1.79) | 6.28 (1.75) | 6.16 (1.79) | 6.22 (1.69) | 6.16 (1.54) | 1 |
| HbA1c [%] | 5.47 (0.31) | 5.45 (0.32) | 5.49 (0.34) | 5.47 (0.30) | 5.46 (0.33) | 1 |
| C-reactive protein [mg/l] # | 1.61 (1.45) | 1.79 (1.80) | 1.76 (1.72) | 1.62 (1.59) | 1.85 (1.74) | 1 |
| Fasting insulin [µlU/ml] # ^1^ | 5.22 (4.77) | 6.03 (6.44) | 6.64 (7.78) | 6.85 (7.40) | 6.56 (6.68) | 0.037 |
| 2-hour insulin [µlU/ml] # ^2^ | 62.69 (48.76) | 63.10 (59.01) | 65.42 (49.82) | 58.51 (46.05) | 62.59 (48.57) | 1 |
| HOMA-IR # ^1^ | 1.28 (1.24) | 1.47 (1.77) | 1.61 (2.00) | 1.66 (1.92) | 1.60 (1.72) | 0.090 |
| Cholesterol [mmol/l] # | 5.84 (0.93) | 5.82 (1.02) | 5.69 (0.96) | 5.79 (1.05) | 5.84 (1.05) | 1 |
| Triglycerides [mmol/l] # | 1.47 (0.92) | 1.54 (0.91) | 1.34 (0.79) | 1.41 (0.88) | 1.47 (1.40) | 1 |
| Systolic blood pressure [mm Hg] | 124.27 (18.32) | 123.63 (18.55) | 123.74 (19.11) | 121.97 (17.03) | 123.03 (18.08) | 1 |
| Diastolic blood pressure [mm Hg] | 76.66 (10.09) | 76.21 (10.09) | 76.32 (9.80) | 75.66 (9.98) | 76.29 (9.45) | 1 |
| CD8^+^ T cells # | 0.10 (0.07) | 0.10 (0.06) | 0.10 (0.07) | 0.11 (0.06) | 0.09 (0.07) | 1 |
| CD4^+^ T cells | 0.17 (0.06) | 0.16 (0.06) | 0.17 (0.06) | 0.16 (0.06) | 0.17 (0.06) | 1 |
| Natural killer cells # | 0.03 (0.03) | 0.03 (0.02) | 0.02 (0.02) | 0.02 (0.03) | 0.03 (0.02) | 1 |
| B cells # | 0.05 (0.04) | 0.05 (0.02) | 0.05 (0.02) | 0.05 (0.02) | 0.05 (0.02) | 1 |
| Monocytes | 0.12 (0.02) | 0.12 (0.02) | 0.12 (0.03) | 0.12 (0.02) | 0.12 (0.03) | 1 |
| Granulocytes | 0.62 (0.09) | 0.63 (0.09) | 0.62 (0.09) | 0.63 (0.08) | 0.64 (0.09) | 0.054 |
| **Categorial phenotypes** | **number** | **number (p-value)** | **number (p-value)** | **number (p-value)** | **number (p-value)** | **-** |
| sex [male/female] | 134/153 | 148/139 (0.312) | 125/162 (0.414) | 134/153 (1.000) | 135/153 (1.000) | - |
| glucose status [combination of IFG and IGT/IFG/IGT/NGT] | 15/14/40/218 | 8/10/41/228 (0.423) | 10/17/45/215 (0.661) | 6/13/49/219 (0.190) | 10/17/33/228 (0.686) | - |

Means, standard deviations and p-values for trend are presented for the different quintiles for the continuous phenotypes. For the categorical variables total numbers of individuals in the different quintiles and p-values for the comparison of the corresponding quintile vs the quintile 1 are given.

# variables were log transformed for determination of p-values

* p-values are still significant after Bonferroni adjustment

+ Proportions of cell types were estimated using method developed by Houseman *et al.* (1)

^1^ Variable only available for 1,440 samples, distribution between the quintiles (286/285/286/285/286)

^2^ Variable only available for 617 samples, distribution between the quintiles (123/123/123/123/123)

IFG: impaired fasting glucose

IGT: impaired glucose tolerance

NGT, normal glucose tolerance

**Reference**

1. Houseman EA, Accomando WP, Koestler DC, Christensen BC, Marsit CJ, Nelson HH, et al. DNA methylation arrays as surrogate measures of cell mixture distribution. BMC Bioinformatics. 2012;13:86.
