# Supplementary material for: Association between DNA Methylation in Whole Blood and Measures of Glucose Metabolism: KORA F4 Study
Source: PLoS One. 2016 Mar 28;11(3):e0152314. doi: 10.1371/journal.pone.0152314 (PMC4809492; doi:10.1371/journal.pone.0152314)
Supplement: S14 Table — (DOC) [file pone.0152314.s014.doc]

**S14 Table. Summary of the analysis for association between DNA methylation and gene expression showing the top association per CpG sites for unadjusted p-values < 0.05.**

| **CpG site** | **transcript** | **Coefficient** | **p-value** | **B-H-adj. p-value** | **annotated gene for CpG site** | **annotated gene for transcript** |
| --- | --- | --- | --- | --- | --- | --- |
| **cg00574958** | **ILMN_1744835** | -3.416 | 4.41x10^-3^ | 0.330 | *CPT1A* | *MRPL21* |
| **cg06500161** | **ILMN_2329927** | -3.646 | 1.94x10^-12^ | 1.50x10^-9^ | *ABCG1* | *ABCG1* |
| **cg11024682** | **ILMN_2060770** | -1.022 | 7.97x10^-3^ | 0.474 | *SREBF1* | *RAI1* |
| **cg22040809** | **ILMN_1700067** | -1.223 | 1.54x10^-3^ | 0.155 | *HCG11* | *BTN3A2* |
| **cg03581271** | **ILMN_1813400** | 0.825 | 0.023 | 0.664 | *PALLD* | *CBR4* |
| **cg09613192** | **ILMN_2390338** | -0.659 | 1.60x10^-3^ | 0.155 | unknown | *UBE2E3* |
| **cg09694782** | **ILMN_1769752** | 0.884 | 1.07x10^-3^ | 0.155 | unknown | *FER1L5* |
| **cg11376147** | **ILMN_2233099** | 1.289 | 0.010 | 0.535 | *SLC43A1* | *SSRP1* |
| **cg17266233** | **ILMN_1660882** | -1.204 | 0.033 | 0.664 | *DGKZ* | *CHRM4* |
| **cg22065733** | **ILMN_2144088** | -3.110 | 1.37x10^-3^ | 0.155 | unknown | *FDFT1* |
| **cg23899654** | **ILMN_2144088** | -4.139 | 0.021 | 0.657 | unknown | *FDFT1* |
| **cg04161365** | **ILMN_1716441** | 0.983 | 0.019 | 0.648 | *DHRS13* | *SNORD4A* |
| **cg20477259** | **ILMN_2150787** | -1.974 | 8.53x10^-4^ | 0.155 | *TNF* | *HLA-C,* |
| **cg01751800** | **ILMN_1691942** | 0.410 | 0.046 | 0.666 | *ANKRD56* | *CCNI* |
| **cg02711608** | **ILMN_2083595** | 0.469 | 0.014 | 0.567 | *SLC1A5* | unknown |
| **cg03979241** | **ILMN_1802971** | 0.598 | 0.042 | 0.666 | *EPB49* | *FAM160B2* |
| **cg06946797** | **ILMN_1790537** | -0.550 | 0.013 | 0.567 | unknown | *RMI2* |
| **cg09349128** | **ILMN_1751471** | -0.734 | 5.06x10^-3^ | 0.330 | unknown | *MLC1* |
| **cg09469355** | **ILMN_1794742** | 0.457 | 0.032 | 0.664 | *SKI* | *HES5* |
| **cg11307565** | **ILMN_1697529** | 2.368 | 0.025 | 0.664 | *PXN* | *RNF10* |
| **cg11990813** | **ILMN_1790896** | 0.805 | 0.032 | 0.664 | *KIAA0664* | *LOC284009* |
| **cg12593793** | **ILMN_1717934** | -1.990 | 5.51x10^-4^ | 0.142 | unknown | *SYT11* |
| **cg22798214** | **ILMN_1830362** | 0.442 | 0.019 | 0.648 | unknown | *NSL1* |

The coefficient and unadjusted as well as B-H-adjusted p-value are presented. Furthermore, the annotated genes for the CpG site and the transcript are listed.
