# Supplementary material for: Association between DNA Methylation in Whole Blood and Measures of Glucose Metabolism: KORA F4 Study
Source: PLoS One. 2016 Mar 28;11(3):e0152314. doi: 10.1371/journal.pone.0152314 (PMC4809492; doi:10.1371/journal.pone.0152314)
Supplement: S16 Table — The table gives p-values corrected using the Benjamini-Hochberg method for multiple testing and the ratio of the number of genes uploaded in the software/total number of genes included in the pathway are presented for each pathway. Underlined pathways are significant after correction for multiple testing using Benjamini-Hochberg. (DOC) [file pone.0152314.s016.doc]

**S16 Table. Pathway analysis based on the top 1,000 CpG sites associated with 2-hour glucose (for results from model 2).**

| **Ingenuity Canonical Pathways** | **B-H-adj. p-value** | **Ratio** |
| --- | --- | --- |
| Thyroid Hormone Metabolism II (via Conjugation and/or Degradation) | 3.78x10^-3^ | 8/29 |
| Serotonin Degradation | 0.052 | 9/56 |
| Nicotine Degradation III | 0.052 | 8/47 |
| Melatonin Degradation I | 0.0581 | 8/50 |
| Nicotine Degradation II | 0.0694 | 8/53 |
| Superpathway of Melatonin Degradation | 0.0743 | 8/55 |
| Estrogen-mediated S-phase Entry | 0.109 | 5/24 |
| Growth Hormone Signaling | 0.195 | 8/69 |
| Adipogenesis pathway | 0.195 | 12/132 |
| Wnt/Ca+ pathway | 0.195 | 7/55 |

The table gives p-values corrected using the Benjamini-Hochberg method for multiple testing and the ratio of the number of genes uploaded in the software/total number of genes included in the pathway are presented for each pathway. Underlined pathways are significant after correction for multiple testing using Benjamini-Hochberg.
