# Supplementary material for: Association between DNA Methylation in Whole Blood and Measures of Glucose Metabolism: KORA F4 Study
Source: PLoS One. 2016 Mar 28;11(3):e0152314. doi: 10.1371/journal.pone.0152314 (PMC4809492; doi:10.1371/journal.pone.0152314)
Supplement: S17 Table — The table gives p-values corrected using the Benjamini-Hochberg method for multiple testing and the ratio of the number of genes uploaded in the software/total number of genes included in the pathway are presented for each pathway. (DOC) [file pone.0152314.s017.doc]

**S17 Table. Pathway analysis based on the top 1,000 CpG sites associated with fasting insulin (for results from model 1).**

| **Ingenuity Canonical Pathways** | **B-H-adj. p-value** | **Ratio** |
| --- | --- | --- |
| Hypoxia Signaling in the Cardiovascular System | 0.287 | 8/63 |
| Reelin Signaling in Neurons | 0.287 | 9/79 |
| PAK Signaling | 0.287 | 9/88 |
| NGF Signaling | 0.287 | 10/106 |
| Role of Tissue Factor in Cancer | 0.287 | 10/107 |
| Glioblastoma Multiforme Signaling | 0.287 | 12/145 |
| Estrogen-Dependent Breast Cancer Signaling | 0.287 | 7/63 |
| Superpathway of Inositol Phosphate Compounds | 0.287 | 14/186 |
| CD40 Signaling | 0.287 | 7/64 |
| B Cell Receptor Signaling | 0.287 | 13/171 |

The table gives p-values corrected using the Benjamini-Hochberg method for multiple testing and the ratio of the number of genes uploaded in the software/total number of genes included in the pathway are presented for each pathway.
