# Supplementary material for: Association between DNA Methylation in Whole Blood and Measures of Glucose Metabolism: KORA F4 Study
Source: PLoS One. 2016 Mar 28;11(3):e0152314. doi: 10.1371/journal.pone.0152314 (PMC4809492; doi:10.1371/journal.pone.0152314)
Supplement: S18 Table — The table gives p-values corrected using the Benjamini-Hochberg method for multiple testing and the ratio of the number of genes uploaded in the software/total number of genes included in the pathway are presented for each pathway. Underlined pathways are significant after correction for multiple testing using Benjamini-Hochberg. (DOC) [file pone.0152314.s018.doc]

**S18 Table. Pathway analysis based on the top 1,000 CpG sites associated with fasting insulin (for results from model 2).**

| **Ingenuity Canonical Pathways** | **B-H-adj. p-value** | **Ratio** |
| --- | --- | --- |
| Wnt/Ca+ pathway | 9.05x10^-3^ | 10/55 |
| Netrin Signaling | 0.0592 | 7/39 |
| B Cell Receptor Signaling | 0.0592 | 16/171 |
| Role of Macrophages, Fibroblasts and Endothelial Cells in Rheumatoid Arthritis | 0.069 | 22/287 |
| ATM Signaling | 0.0773 | 8/59 |
| Calcium Signaling | 0.0773 | 15/170 |
| Huntington's Disease Signaling | 0.0773 | 18/226 |
| G-Protein Coupled Receptor Signaling | 0.091 | 19/254 |
| Reelin Signaling in Neurons | 0.091 | 9/79 |
| ILK Signaling | 0.091 | 15/181 |

The table gives p-values corrected using the Benjamini-Hochberg method for multiple testing and the ratio of the number of genes uploaded in the software/total number of genes included in the pathway are presented for each pathway. Underlined pathways are significant after correction for multiple testing using Benjamini-Hochberg.
