# Supplementary material for: Association between DNA Methylation in Whole Blood and Measures of Glucose Metabolism: KORA F4 Study
Source: PLoS One. 2016 Mar 28;11(3):e0152314. doi: 10.1371/journal.pone.0152314 (PMC4809492; doi:10.1371/journal.pone.0152314)
Supplement: S19 Table — The table gives p-values corrected using the Benjamini-Hochberg method for multiple testing and the ratio of the number of genes uploaded in the software/total number of genes included in the pathway are presented for each pathway. (DOC) [file pone.0152314.s019.doc]

**S19 Table. Pathway analysis based on the top 1,000 CpG sites associated with HOMA-IR (for results from model 1).**

| **Ingenuity Canonical Pathways** | **B-H-adj. p-value** | **Ratio** |
| --- | --- | --- |
| Hypoxia Signaling in the Cardiovascular System | 0.368 | 8/63 |
| Reelin Signaling in Neurons | 0.368 | 9/79 |
| Role of Tissue Factor in Cancer | 0.368 | 10/107 |
| IL-15 Signaling | 0.368 | 7/63 |
| Estrogen-Dependent Breast Cancer Signaling | 0.368 | 7/63 |
| ERK/MAPK Signaling | 0.368 | 14/186 |
| Renal Cell Carcinoma Signaling | 0.368 | 7/69 |
| FAK Signaling | 0.368 | 8/86 |
| Integrin Signaling | 0.368 | 14/200 |
| FLT3 Signaling in Hematopoietic Progenitor Cells | 0.368 | 7/71 |

The table gives p-values corrected using the Benjamini-Hochberg method for multiple testing and the ratio of the number of genes uploaded in the software/total number of genes included in the pathway are presented for each pathway.
