# Supplementary material for: Association between DNA Methylation in Whole Blood and Measures of Glucose Metabolism: KORA F4 Study
Source: PLoS One. 2016 Mar 28;11(3):e0152314. doi: 10.1371/journal.pone.0152314 (PMC4809492; doi:10.1371/journal.pone.0152314)
Supplement: S20 Table — The table gives p-values corrected using the Benjamini-Hochberg method for multiple testing and the ratio of the number of genes uploaded in the software/total number of genes included in the pathway are presented for each pathway. Underlined pathways are significant after correction for multiple testing using Benjamini-Hochberg. (DOC) [file pone.0152314.s020.doc]

**S20 Table. Pathway analysis based on the top 1,000 CpG sites associated with HOMA-IR (for results from model 2).**

| **Ingenuity Canonical Pathways** | **B-H-adj. p-value** | **Ratio** |
| --- | --- | --- |
| Wnt/Ca+ pathway | 0.0482 | 9/55 |
| ILK Signaling | 0.0846 | 16/181 |
| ATM Signaling | 0.0846 | 8/59 |
| B Cell Receptor Signaling | 0.0846 | 15/171 |
| Ephrin Receptor Signaling | 0.0846 | 15/172 |
| Actin Cytoskeleton Signaling | 0.0846 | 17/210 |
| Reelin Signaling in Neurons | 0.0846 | 9/79 |
| Netrin Signaling | 0.0846 | 6/39 |
| Agrin Interactions at Neuromuscular Junction | 0.0846 | 8/67 |
| Neurotrophin/TRK Signaling | 0.0846 | 8/67 |

The table gives p-values corrected using the Benjamini-Hochberg method for multiple testing and the ratio of the number of genes uploaded in the software/total number of genes included in the pathway are presented for each pathway. Underlined pathways are significant after correction for multiple testing using Benjamini-Hochberg.
