# Supplementary material for: Association between DNA Methylation in Whole Blood and Measures of Glucose Metabolism: KORA F4 Study
Source: PLoS One. 2016 Mar 28;11(3):e0152314. doi: 10.1371/journal.pone.0152314 (PMC4809492; doi:10.1371/journal.pone.0152314)
Supplement: S21 Table — Informations were taken from www.ncbi.nlm.nih.gov/gene/ in February 2016, if not stated otherwise. (DOC) [file pone.0152314.s021.doc]

**S21 Table. Function of genes showing significant associations between methylation status and parameters of glucose metabolism.**

| **Gene** | **Function** |
| --- | --- |
| *ABCG1* | ATP-binding cassette, sub-family G (WHITE), member 1  Encodes a protein belonging to the superfamily of ATP-binding cassette (ABC) transporters which transport different molecules across extra- and intra-cellular membranes. *ABCG1* is involved in macrophage cholesterol and phospholipids transport and is assumed to regulate cellular lipid homeostasis on other cell types. |
| *ANKRD56* | Ankyrin Repeat Domain-Containing Protein 56 [synonym of SOWAHB (sosondowah ankyrin repeat domain family member B)]  No known function. |
| *CPT1A* | Carnitine palmitoyltransferase 1a  CPT1A is an isoform of carnitine palmitoyltransferase (CPT) I. By the sequential action of CPT and carnitine palmitoyltransferase II, together with a carnitine-acylcarnitine translocase, the mitochondrial oxidation of long-chain fatty acids is initiated. CPT I is the key enzyme in the carnitine-dependent transport across the mitochondrial inner membrane. Deficiency of CPT I leads to a reduced rate of fatty acid beta-oxidation. |
| *CREB3L2* | cAMP responsive element binding protein 3-like 2  Encodes a member of the oasis bZIP transcription factor family. The encoded protein is a transcriptional activator. |
| *DHRS13* | Dehydrogenase/reductase (SDR family) member 13  No known function. |
| *DKGZ* | Diacylglycerol kinase zeta  Encodes a protein belonging to the eukaryotic diacylglycerol kinase family. It can reduce protein kinase C activity by regulating intracellular diacylglycerol levels. |
| *EPB49* | Erythrocyte membrane protein band 4.9 (dematin)  No known function. |
| *ESCO1* | Establishment of sister chromatid cohesion N-acetyltransferase 1  Belongs to a conserved family of acetyltransferases involved in sister chromatid cohesion (1) |
| *HCG11* | HLA complex group 11 (non-protein coding)  No known function. |
| *KIAA0664* | Synonym of CLUH (Clustered mitochondria protein homolog)  No known function. |
| *PALLD* | Palladin, cytoskeletal associated protein  Encodes a cytoskeletal protein needed for organizing the actin cytoskeleton. It is involved in cell shape, adhesion, and contraction. Polymorphisms in this gene are associated with increased susceptibility to pancreatic cancer type 1 and risk for myocardial infarction. |
| *PXN* | Paxillin  Encodes a cytoskeletal protein involved in actin-membrane attachment at sites of cell adhesion to the extracellular matrix. |
| *SKI* | SKI proto-oncogene  Encodes the nuclear protooncogene protein homolog of avian sarcoma viral oncogene. It inhibits the TGF-beta signaling, and may be involved in neural tube development and muscle differentiation. |
| *SLC1A5* | Solute carrier family 1 (neutral amino acid transporter), member 5  Encodes a sodium-dependent neutral amino acid transporter that can act as a receptor for RD114/type D retrovirus (2). |
| *SLC43A1* | Solute carrier family 43 (amino acid system L transporter) member 1  Belongs to the system L family of plasma membrane carrier proteins that transport large neutral amino acids (3). |
| *SREBF1* | Sterol regulatory element binding transcription factor 1  Encodes a transcription factor binding to the sterol regulatory element-1, which is a decamer flanking the low density lipoprotein receptor gene and some genes involved in sterol biosynthesis. It is synthesized as a precursor attached to the nuclear membrane and endoplasmic reticulum. |
| *STK40* | Serine/threonine kinase 40  Belongs to the serine/threonine kinase and is essential in diverse signaling pathways associated with a wide range of cellular activities, including proliferation, differentiation, survival, and apoptosis (4). It was shown to induce extraembyonic endoderm differentiation from mouse embryonic stem cells (5). |
| *TNF* | Tumor necrosis factor  Encodes a multifunctional proinflammatory cytokine belonging to the tumor necrosis factor superfamily. It is mainly secreted by macrophages. The cytokine is involved in the regulation of different biological processes including cell proliferation, differentiation, apoptosis, lipid metabolism, and coagulation. Furthermore, it is implicated in autoimmune diseases, insulin resistance, and cancer. |

Informations were taken from www.ncbi.nlm.nih.gov/gene/ on February 2016, if not stated otherwise.

**References**

1. Hou F, Zou H. Two human orthologues of Eco1/Ctf7 acetyltransferases are both required for proper sister-chromatid cohesion. Mol Biol Cell. 2005;16(8):3908-18.

2. Larriba S, Sumoy L, Ramos MD, Gimenez J, Estivill X, Casals T, et al. ATB(0)/SLC1A5 gene. Fine localisation and exclusion of association with the intestinal phenotype of cystic fibrosis. Eur J Hum Genet. 2001;9(11):860-6.

3. Babu E, Kanai Y, Chairoungdua A, Kim DK, Iribe Y, Tangtrongsup S, et al. Identification of a novel system L amino acid transporter structurally distinct from heterodimeric amino acid transporters. J Biol Chem. 2003;278(44):43838-45.

4. Zhang J, Zhang J, Zhao C, Shen R, Guo X, Li C, et al. Analysis of transcription factor Stk40 expression and function during mouse pre-implantation embryonic development. Molecular medicine reports. 2014;9(2):535-40.

5. Li L, Sun L, Gao F, Jiang J, Yang Y, Li C, et al. Stk40 links the pluripotency factor Oct4 to the Erk/MAPK pathway and controls extraembryonic endoderm differentiation. Proc Natl Acad Sci U S A. 2010;107(4):1402-7.
